# Harnessing the power of *Arctium lappa root*: a review of its pharmacological properties and therapeutic applications

**DOI:** 10.1007/s13659-024-00466-8

**Published:** 2024-08-20

**Authors:** Mukul Shyam, Evan Prince Sabina

**Affiliations:** Department of Biotechnology, School of Biosciences and Technology, VIT University, SBST, VIT, Vellore, 632014 Tamil Nadu India

**Keywords:** Antioxidant, Anti-diabetic, Burdock root, Phytoconstituents, Traditional medicine

## Abstract

*Arctium lappa,* widely recognized as burdock, is a perennial plant that is employed in the realm of traditional Chinese medicine for a wide range of medicinal applications. The herb is rich in bioactive metabolites with therapeutic potential, encompassing polyphenolic antioxidants in its leaves, and flavonoids and fructo-oligosaccharides in its underground parts. Nutraceuticals originating from botanical sources such as *Arctium lappa* provide supplementary health advantages alongside their nutritional content and have demonstrated effectiveness in the prevention and management of specific ailments. The utilization of *Arctium lappa* root extract has exhibited encouraging outcomes in addressing hepatotoxicity induced by cadmium, lead, chromium, and acetaminophen, ameliorating liver damage and oxidative stress. Additionally, the root extract displays properties such as antidiabetic, hypolipidemic, aphrodisiac, anti-rheumatic, anti-Alzheimer, and various other pharmacological actions.

**Graphical abstract:**

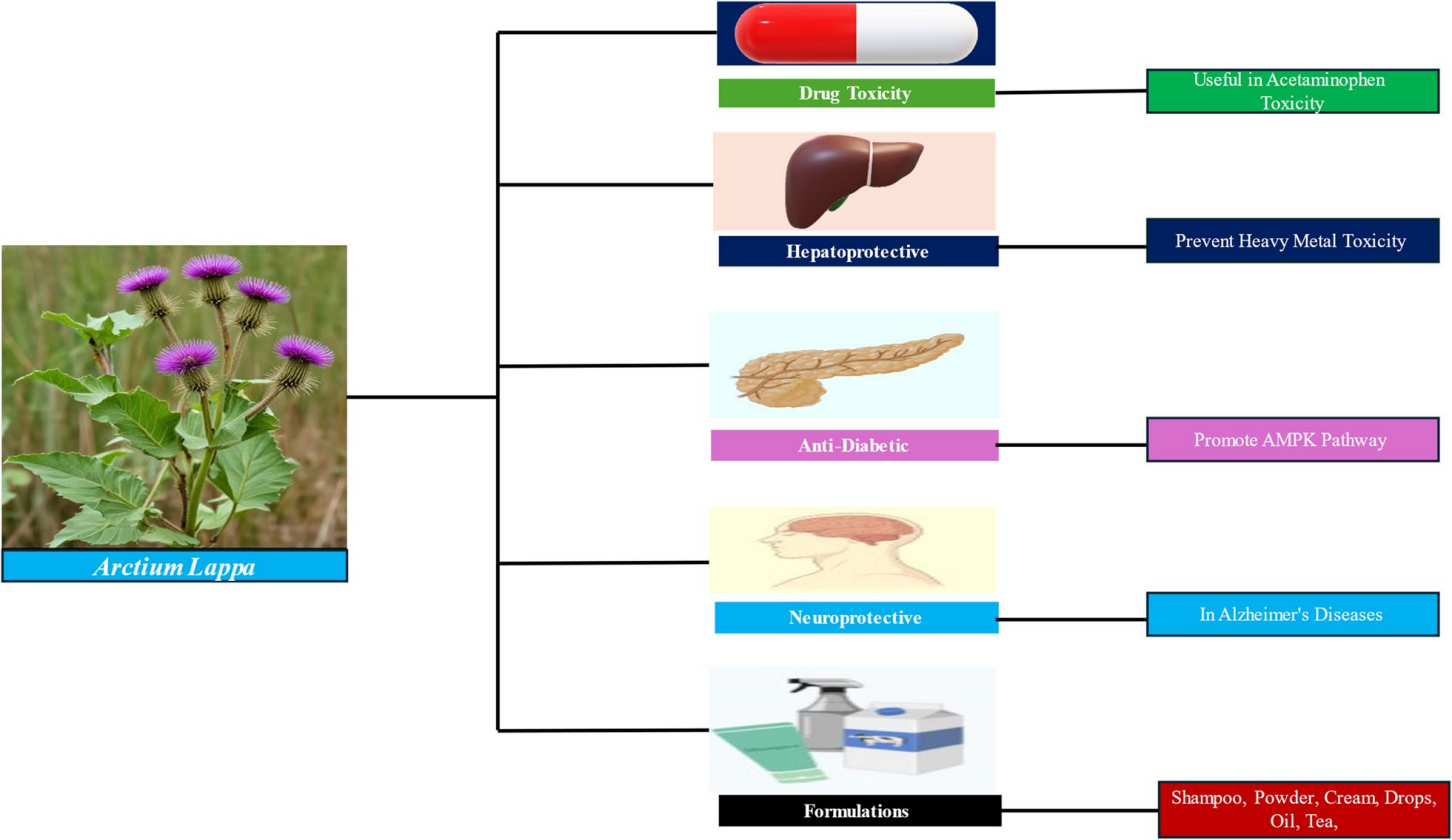

## Introduction

*Arctium lappa* (*A. lappa L.*) is classified as a perennial herb with a rich historical background, demonstrating longevity in its usage, and is widely recognized by the name burdock or bardana. *A. lappa L.* has been an integral part of traditional Chinese medicine for numerous centuries, with its roots, fruits, seeds, and leaves being utilized in a diverse array of medicinal applications. The plant houses numerous bioactive metabolites, each exhibiting noteworthy therapeutic potential. Burdock foliage has been discovered to possess a substantial content of polyphenolic antioxidants and exhibits robust antioxidant attributes. The most effective approach for obtaining a burdock leaf extract with elevated levels of polyphenolic compounds entails employing freeze-drying, succeeded by an ethanol extraction ranging from 30 to 50% [1]. Moreover, the efficacy of burdock leaf extract has been evidenced through significant antioxidant capabilities, including the capacity to eliminate superoxide anions and counteract hydroxyl radicals. The incorporation of burdock leaf extract in skin care products is widespread owing to its anti-aging and skin-smoothing attributes [2]. Wherein the seeds of the burdock plant encompass biologically active compounds, such as flavonoids and fructo-oligosaccharides, which contribute to its pharmacological properties [3]. Furthermore, it has been demonstrated that burdock seeds possess diuretic qualities. Moreover, it has been observed that burdock seed extract, enriched with arctigenin, exhibits skin-brightening capabilities, and can enhance the evenness of skin tone. Burdock root is regarded as superior to burdock seed and fruit due to its elevated nutritional value and potential health advantages. The foundation of the burdock plant serves as a storage of prebiotic fibers, chlorogenic acids, cinnarine, lignans, and quercetin, demonstrating encouraging antioxidant, anti-inflammatory, and hypolipidemic characteristics. Overall, burdock roots exhibit potential health advantages and can be utilized for diverse therapeutic purposes. The extract derived from burdock root presents antidiabetic, hypolipidemic, and hepatoprotective properties in the context of nicotinamide-streptozotocin-induced type-2 diabetes. In addition, it impedes the formation of the end-glycosylated product, thereby contributing to the management of complications associated with diabetes [4, 5]. The root of *A. lappa L.* exhibits a range of pharmacological effects, such as anti-cancer, neuroprotection, anti-rheumatic, and aphrodisiac, among others, as detailed in the present review article.

## Cultivation of *A. lappa L.*

**Fig. 1:**
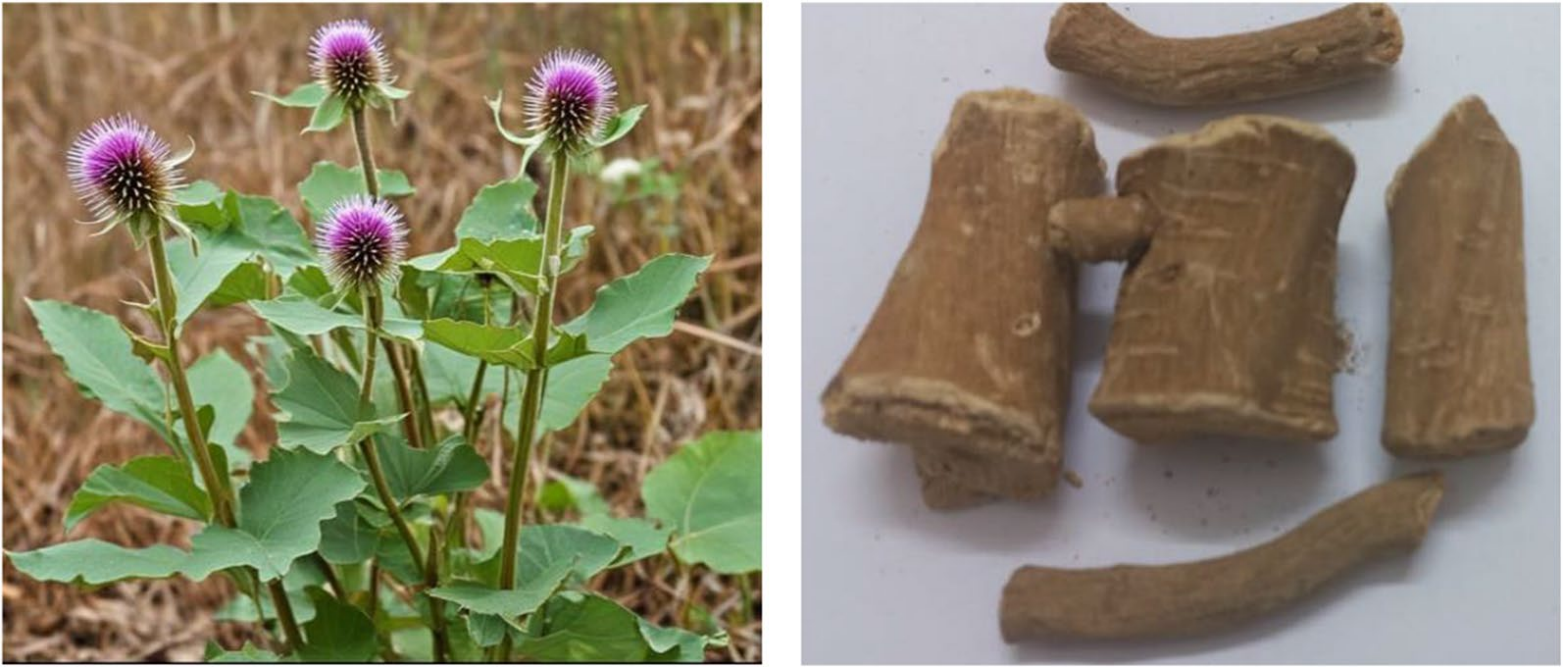
Schematic depiction of *Arctium lappa L*. belongs to the Plantae kingdom, Tracheophyte phylum, Magnoliopsida class, Asterales order, Asteraceae family, and Arctium genus [7]

*A. lappa L.* is a plant that completes its life cycle in 2 years, reaching a height of ~ 1 m. The initial year is characterized by the presence of lengthy, tapered roots, complemented by an upright stem adorned with large, oval-shaped leaves. The second year, on the other hand, showcases smaller leaves distributed along hairy and creased branches. The undersides of these leaves are adorned with a layer of white hairs. *A. lappa L.* generates globular terminal floral heads measuring around 4 cm, solely comprising purplish tubular flowers. The fruit produced is a three-sided achene [6] (Fig. 1)

The extensive geographic distribution of *A. lappa L.* encompasses various regions worldwide, manifesting diverse color variations ranging from white to yellowish white upon the plant’s attainment of maturity. The identification of the *A. lappa L.* plant is characterized by a diversity of vernacular names, reflecting its regional origins. For example, it is referred to as Niubang in China, known as gobo in Japanese, and recognized as repejnik in Russia [8].

## Nutraceutical property of *A. lappa L.*

**Fig. 2:**
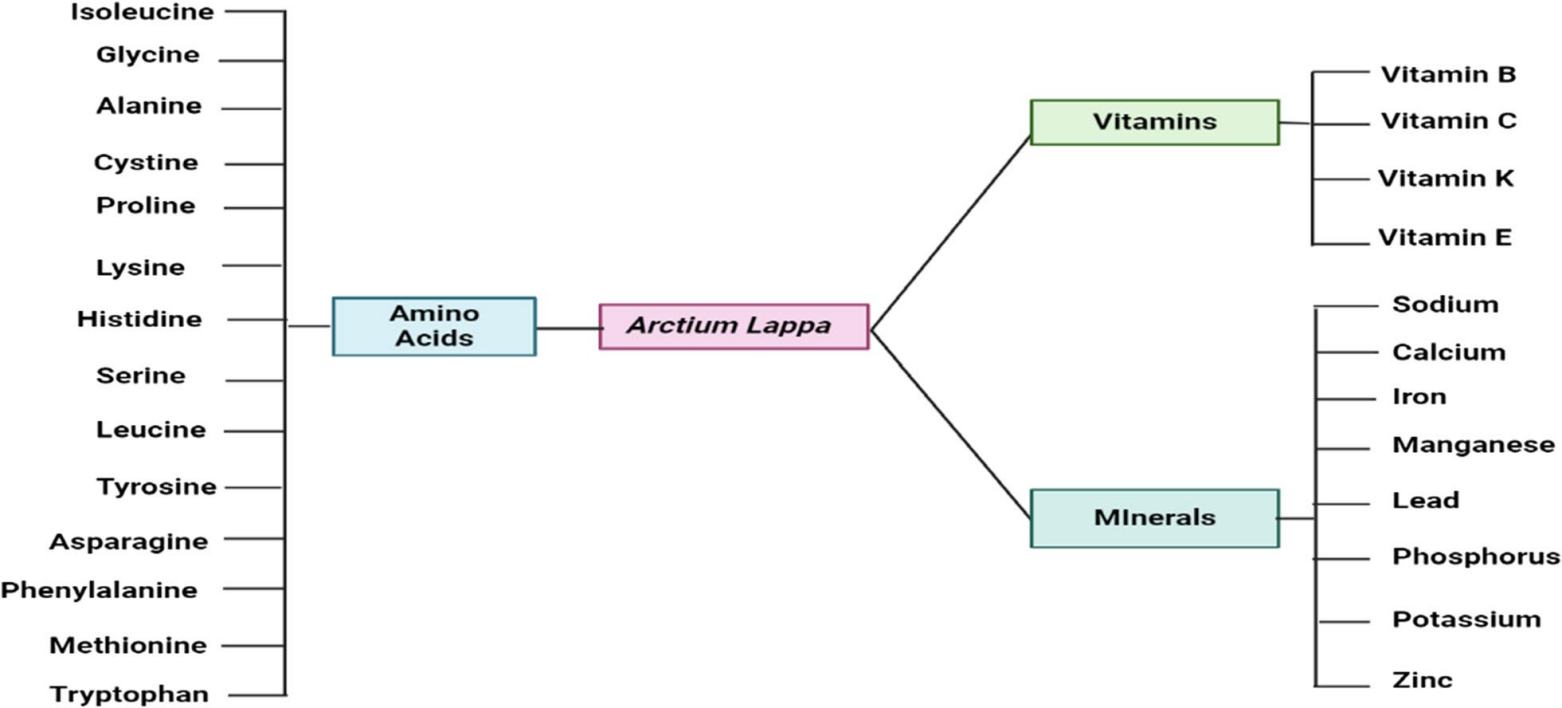
Schematic representation of *A. lappa L.* containing nutrients essential for human health

Nutraceuticals, a class of products that offer supplementary health benefits along with their nutritional value, can be derived from either plants or animals and are administered in a pharmaceutical form. These nutraceuticals possess proven clinical efficacy and can be employed for the prevention and support of specific diseases [9]. Throughout ancient times, the burdock plant has been utilized as a nutraceutical due to its remarkable antioxidant properties. The broad spectrum of activities witnessed in this phenomenon can be ascribed to the existence of multiple active components. These active constituents include tannins, polyphenols like caffeic acid and chlorogenic acid, beta-endemol, dietary fiber such as inulin and lignan (for instance, arctigenin and arctiin), sterols (such as sitosterol and stigmasterol), diarctigenin, essential oils, a multitude of vitamins as well as an array of minerals and amino acids discussed in Fig. 2.

These components are abundantly present throughout the entire plant, although the roots exhibit a higher concentration of these bioactive constituents [10–12]. The components found in burdock roots, seeds, and leaves are outlined in Table 1, including their structure, chemical formula, and the part where they are predominantly found.

**Table 1:** Enumeration of the active components present in Burdock root, seed, and leaves

Category	Constituents	Parts	Chemical formula	Therapeutic activity	IC 50	References
Sterol	Sitosterol-beta-d-glucopyranoside	Roots	C35H60O6	Ameliorate insulin resistance by enhancing insulin signaling molecules like IR and GLUT4 in skeletal muscle, improving glycemic regulation in type-2 diabetic rats	10 µM on L6-GLUT4myc Cell	[13]
Fructose	Inulin	Root	C228H382O191	Inulin enhances glycolipid metabolism through the alteration of the gut microbiota composition and the promotion of fecal bile acid excretion in mice	Inhibit α-glucosidase with an IC50 of 0.4996 mg/ml on α-glucosidase	[14, 15]
Volatile oil	Eugenol	Root	C10H12O2	Anti-caner activity in different types of cancer by inducing apoptosis	200 mg/ml on HeLa cell line	[16, 17]
Polyphenols	Vanillin	Root	C8H8O3	Anti-cancer activity by cell cycle arrest during the G0/G1 phase and an increase in apoptosis in the sub-G0 phase	250 μg/ml on Mel-2 cell line	[18, 19]
	Hesperetin	Root	C16H14O6	Hesperetin reduced neuroinflammation in LPS-induced neuroinflammation by reducing proinflammatory cytokines interleukin (IL)-1β, IL-6, and TNF-α	0.340 mg/ml on MRC-5 fibroblasts cell line	[20, 21]
	Chlorogenic acid (caffeoylquinic acid)	Root, Seed, leaves	C16H18O9	Chlorogenic acid reduces the expression of Keap1 and activates the most sensitive transcription factor erythrocyte derived 2-related factor 2 (Nrf2) signaling pathway and upregulates the mRNA expression of a series of endogenous antioxidant enzymes	Chlorogenic acid 10 µM increased nuclear Nrf2 expression in human umbilical vein endothelial cells	[22]
	Caffeic acid	Root, seed, leaves	C9H8O4	Caffeic acid diminishes oxidative stress and inflammation, enhances insulin sensitivity through the induction of PI3K/Akt signaling, shields against harm induced by advanced glycation end-products, and boosts the abundance of GLUT4 in muscles by triggering AMPK	100 μM of caffeic acid activated AMPK and its downstream target acetyl-CoA-carboxylase in rat skeletal muscle	[23]
	Hesperidin	Root	C28H34O15	Hesperidin lowers cholesterol levels by reducing ACAT1 and ACAT2 activity and upregulates LDL receptors, enhancing lipoprotein reuptake for cholesterol reduction	4.58 µg/ml on HPg2 cell line	[24, 25]
	Cynarin (Dicaffeoylquinic acid)	Root, Seed, leaves	C25H24O12	Cynarin has protective effects on liver damage caused by CCl4, cyclophosphamide, or alcohol	Cynarin 10 ng/ml inhibits p-ERK and p-AKT expression induced by PDGF-BB in CFSC-8B HSC cells	[26, 27]
	Rosmarinic acid	Root	C18H16O8	Rosmarinic Acid Exhibits Anticancer Effects via MARK4(Microtubule affinity regulating kinase) Inhibiting controls the early step of cell division	RA significantly inhibits MARK4 activity at 6204 µM	[28]
Lignans	Lappaol B	Seed	C31H34O9	Lappaol B inhibits LPS-induced NO production	25.9 mM in RAW264.7 cells	[29, 30]
	Arctiin	Root, Seed, leaves	C27H34O11	Arctiin exerts hepatoprotective effects by inhibiting the MAPK pathway, improving lipid accumulation, reducing inflammatory responses, and enhancing oxidative stress capacity	Arctiin dose 50 mg/kg reduces levels of p-ERK/ERK, JNK/p-JNK, and p-P38/P38 in mice for 16 weeks, fed with a high-fat diet	[31, 32]
		Seed	C40H42O12	Lappaol F exerts antitumor effects by activating CDKN1C/p57 in colorectal cancer cells, leading to S phase arrest and proliferation inhibition	42.9 μmol/l PC3 cell line	[33, 34]
	Matairesinol	Seed	C20H22O6	Matairesinol demonstrates anti-inflammatory and antioxidant properties in the context of brain injury induced by sepsis, achieved through the inhibition of the MAPK and NF-κB pathways via the activation of AMPK	20 μM Matairesinol following LPS facilitates AMPK and inhibits MAPK and NF-κB pathways in NSC-34 and BV2 cells	[35]
	Lappaol A	Seed, root	C30H32O9	Lappaol A demonstrates anti-allergic properties through the suppression of the allergic reaction triggered by IgE/Ag-activated mast cells	9.5 μm on LPS-stimulated RAW264.7 cells	[30, 36]
	Lappaol C	Seed	C30H34O10	Lappaol C induces apoptosis in cancer cells and suppresses tumor growth via decreasing tumor tolerance to glucose starvation	8 µg/ml NCaP prostate cancer cell line	[37, 38]
	Arctigenin	Seed, Root	C21H2406	Arctigenin inhibits cell growth, migration, and invasion in hepatocellular carcinoma cells by targeting the gankyrin promoter and recruiting C/EBPα, subsequently leading to decreased gankyrin expression	59.27 Nm on Hep3B cell line	[39]
	Diarctigenin	Root, seeds	C42H46O12	Diarctigenin suppressed the synthesis of nitric oxide, prostaglandin E2, TNF-α, as well as interleukin (IL)-1beta and IL-6, in macrophages activated by zymosan	6–12 μM in zymosan-activated macrophages	[40]
Flavonoid	Rutin	Seed, leaves	C27H30O16	Rutin shows a gastroprotective effect against Indomethacin-induced ulcers in rats by inhibiting neutrophil infiltration, suppressing oxidative stress generation, and replenishing nitrite⁄nitrate levels	Rutin (200 mg⁄kg in rats show anti-oxidant action by restoring reduced glutathione levels	[41]
	Kaempferol	Leaves	C15H10O6	Kaempferol demonstrates hepatoprotective effects through antioxidative and anti-inflammatory mechanisms, inhibiting MAPK/NF-κB signaling and activating AMPK/Nrf2 pathways in liver damage models	Kaempferol 25 mg/kg Inhibit OATP1B1 transporter, maintaining a level of AST, ALT in HEK-293 cell line	[42, 43]
	Apigenin	Leaves	C15H10O5	Apigenin protects against oxidative stress-induced myocardial injury by modulating the SIRT1 signaling pathways	Apigenin 10 μM protects cardiomyocytes from hypoxia-reoxygenation-induced injury in the H9c2 cell line	[44]
	Quercetin	Root, leaves	C15H10O7	Quercetin exerts antibacterial activities against different gram-positive bacteria	Quercetin exerted inhibitory effects against Staphylococcus aureus, and Pseudomonas aeruginosa at MIC of 20 mcg/ml	[45]
	Myricitrin	Root	C15H10O8	Myricitrin demonstrates anti-inflammatory effects by blocking JAKs and STAT-1 activation, leading to reduced ROS production and inflammatory responses	Myricitrin at 100, 200, and 400 μg/ml shows inhibit reactive oxygen species RAW264.7 macrophage cells	[46]
	Luteolin	Seed, leaves	C15H10O6	Luteolin exerts antioxidant effect and neuroprotective effects in Alzheimer’s disease and Huntington’s disease	Luteolin at a concentration of 30–100 µg/ml shows Fe^2+^ chelating properties and potent DPPH radical scavenging ability at 1–100 µg/ml concentration	[47]
	Morin	Leaves	C15H10O7	Morin reverses ketamine-induced schizophrenic-like behaviors by attenuating altered dopaminergic, glutamatergic, 5-hydroxytryptaminergic, and cholinergic neurotransmissions in a brain region-dependent manner	Intraperitoneal injection 100 mg/kg of morin for 14 consecutive days in ketamine-induced schizophrenic-like behaviors	[48]

## *A. lappa* L. root formulations and products

**Fig. 3:**
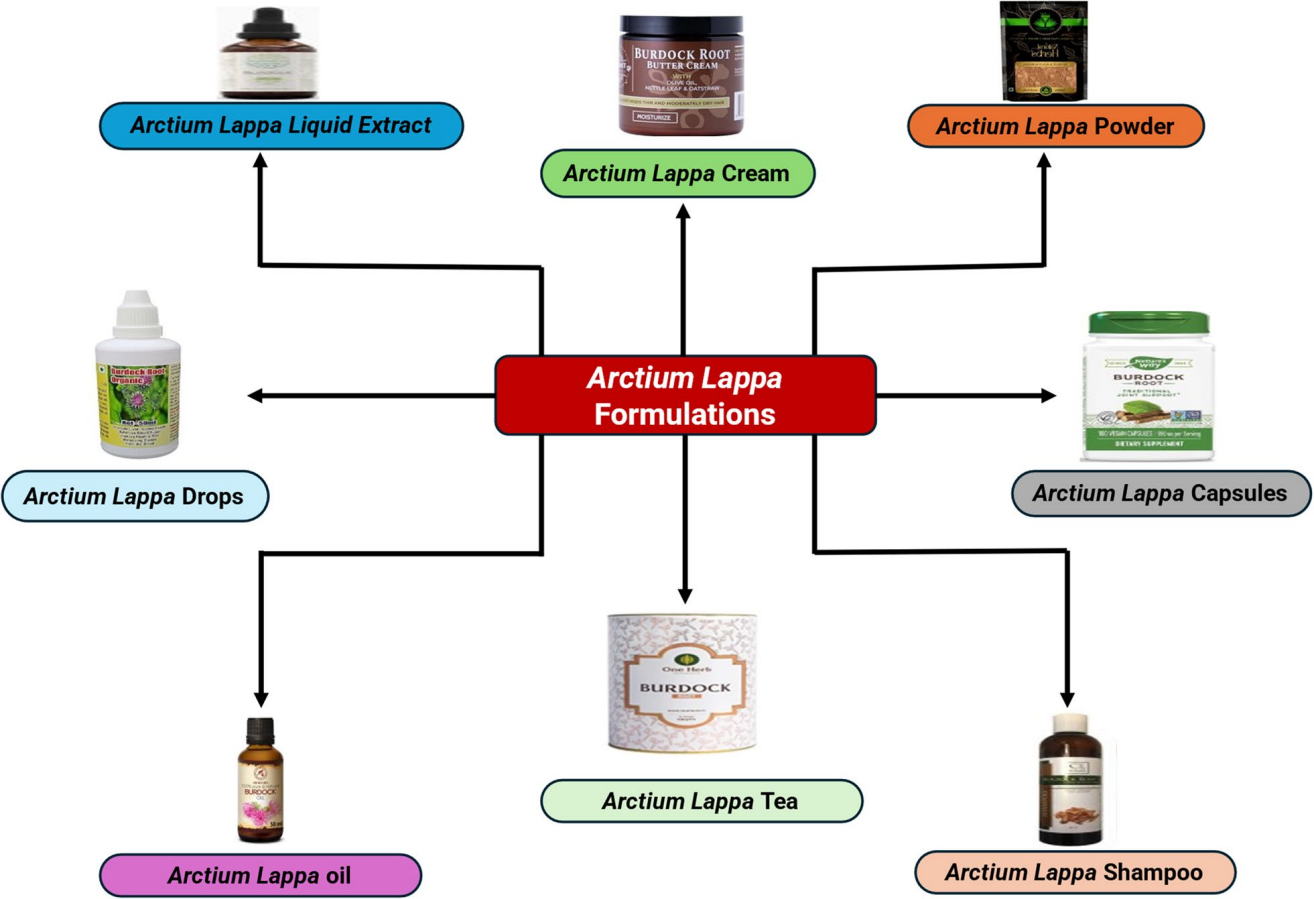
Schematic representation of *A. lappa L*. formulations available in the market

*A. lappa L.* presents a diverse array of formulations that are available in the commercial marketplace. These formulations include a variety of products like *A. lappa L*. root; tea bags, powder, capsules, topical creams and ointments, hair care products, facial cleansers, and masks [49] (Fig. 3).

*A. lappa L*. root flour is employed as a promising prebiotic component in cookies because of its notable abundance of fructo-oligosaccharides and phenolic substances. *A. lappa L*. root flour presents itself as a viable option for imparting phenolic compounds, dietary fibers, and prebiotic oligosaccharides to baked goods, appealing to individuals interested in functional foods that promote well-being [50]. *A. lappa L*. root was also used for the packaging material with the incorporation of cellulose acetate, and it showed improved antimicrobial activity against Aspergillus niger, Candida albicans, Staphylococcus aureus, and Escherichia coli with increasing *A. lappa L*. extract concentration. The film also exhibited increased free radical scavenger activity, total phenol content, elongation to break, contact angle, color difference, and water vapor transmission rate with increasing burdock extract concentration. On the other hand, Young's modulus, tensile strength, solubility, water content, and swelling decreased compared to the control film without burdock extract. Burdock extract-loaded cellulose acetate film was found to be biodegradable and could be used as an active, biodegradable, and environmentally friendly edible film for food packaging [51]. Furthermore, alternative formulations such as *A. lappa L*. root silver and gold nanoparticles also exist, which possess antimicrobial properties against five distinct microorganisms: Escherichia coli (Gram-negative), Agrobacterium tumefaciens (Gram-negative), Lactobacillus acidophilus (Gram-positive), Staphylococcus aureus (Gram-positive), and Trichoderma harzianum (fungus). Additionally, these nanoparticles have demonstrated remarkable catalytic activity in the conversion of pollutants found in wastewater [52].

## Anti-toxicity study of *A. lappa L*. root extract

*A. lappa L*. root extract also helps in the protection and prevention of toxicity. Certain studies have been undertaken to examine the impact of *A. lappa L* root extract on toxicity studies associated with heavy metals and drug-induced toxicity. The activation of *A. lappa L* root extract is predominantly manifested through the prevention of antioxidant substance depletion and inhibition of lipid peroxidation, as elucidated below in the Table 2.

**Table 2:** Anti-toxicity study of *A. lappa L*. phytochemicals

Phytochemical	Heavy metal	Protective mechanism of phytochemical	References
Chlorogenic acid	Cadmium	60 mg/kg body weight by intragastric route restores the activity of AChE in the brain and inhibits lipid peroxidation	[53]
Arctigenin	Cadmium	80 mg/kg of Arctigenin for one week by gastric gavage modulates Nrf2 and NF-κB signaling in cadmium-treated rats and up-regulates the GSH levels and the activity of SOD in Male Wistar rats	[54]
Chlorogenic acid	Lead	100 µM of Chlorogenic acid in zebrafish protects against Pb-induced developmental neurotoxicity by modulating autophagy and restoring the anti-oxidant enzyme	[55]
Chlorogenic acid	Arsenic trioxide	100 mg/kg of chlorogenic acid by intraperitoneal administration in mice decreases serum levels of ALT, AST, and ALP and increases the level of GSH	[56]
Luteolin	Multi-heavy metal mixture	Luteolin 20 µM in HL7702 cells show an inhibitory effect on multi-heavy metal mixture-induced cleavage of caspase-9, caspase-3, and poly(adenosine diphosphate-ribose) polymerase-1 protein	[57]
Luteolin	Cobalt	Luteolin 100 mg/kg was given oral gavage for eight consecutive days in Wistar rats leading to inhibition of apoptosis via the PI3K/AKT pathway, reduction of oxidative stress through activation of haem oxygenase-1, and regulation of MAP kinases and nitric oxide synthases and reduction in Kim-1 expressions	[58]
Luteolin	Lead	50 mg/kg was given daily by oral route for seven days elevating SOD, CAT, GR, GPx, and GSH and lowering the MDA level in hepatic tissue	[59]
Luteolin	Mercury	Kunming mice were treated with luteolin 100 mg/kg 24 h after administration of 4 mg/kg mercuric chloride reversed the changes in levels of inflammation- and apoptosis-related proteins involving NF-κB, TNF-α, Sirt1, mTOR, Bax, p53, and Bcl-2, and inhibited p38 MAPK activation and prevent hepatic injury	[60]
Hesperidin	Cadmium	100 mg/kg of hesperidin for 28 days in Wistar rats by oral route protects cadmium toxicity by reduced elevated LDL-C and Cholesterol while increasing HDL-C and TG	[61]
Hesperetin	Lead	50 mg/kg/day of hesperetin for 8 weeks reduces ALT, AST, urea, uric acid, and creatinine in the serum of lead-treated rats and protects lead-induced liver and nephrotoxicity	[62]
Quercetin	Cadmium	Cd-induced toxicity in the goat testis in vitro in a dose-(10, 50, and 100 μM) decreased apoptotic attributes and restored CAT, SOD, and GST activity	[63]
Apigenin	Arsenic	Apigenin on PC12 cell malondialdehyde, nitric oxide, and ROS in addition to the enzymatic and non-enzymatic antioxidant molecules such as catalase, glutathione, and superoxide dismutase were assessed	[64]

### Cadmium toxicity

**Fig. 4:**
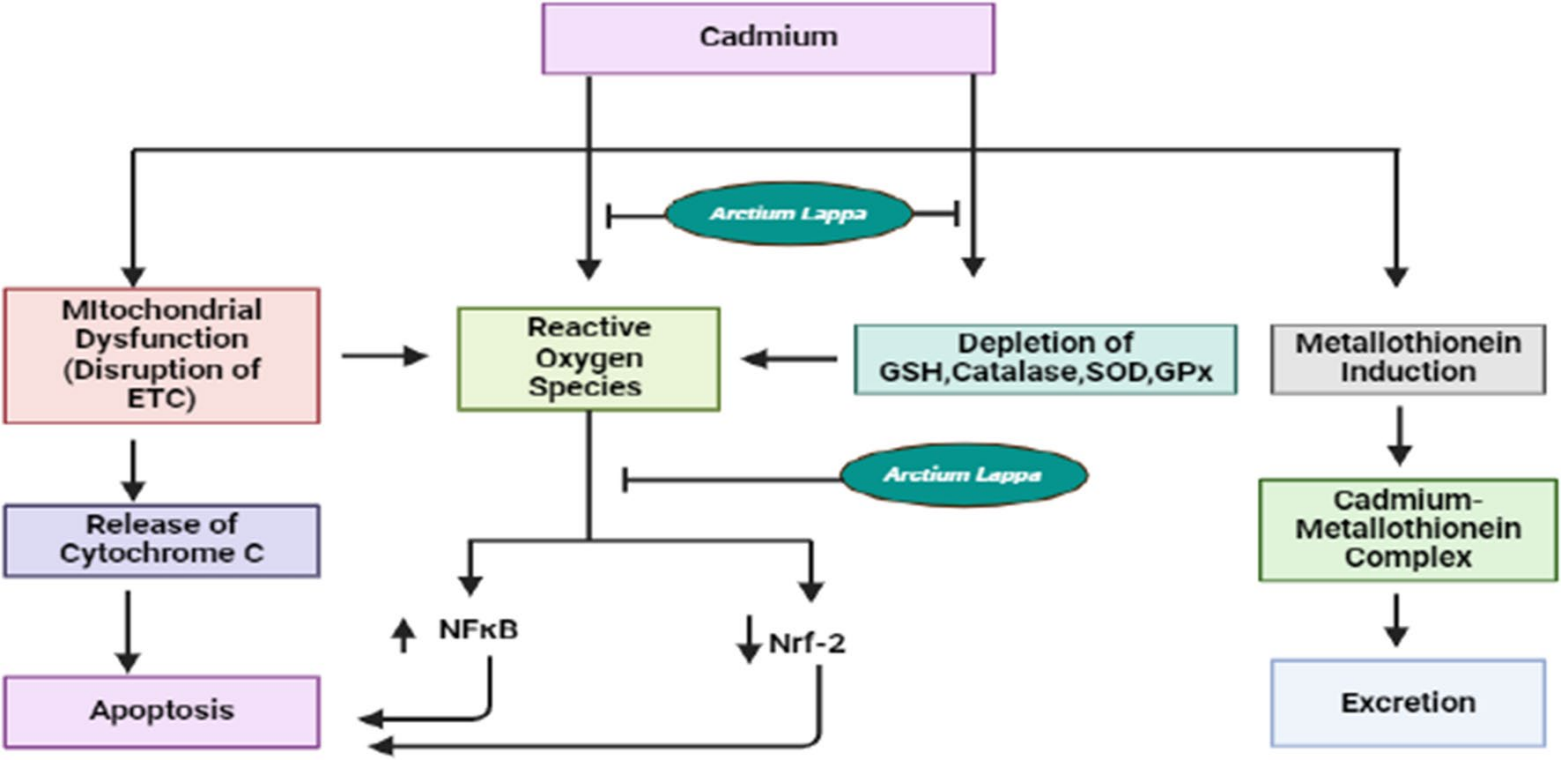
Schematic representation of cadmium causing toxicity. This illustration depicts how cadmium induces toxicity through various mechanisms, such as the generation of reactive oxygen species, disruption of the Electron Transport Chain (ETC), and depletion of antioxidant enzymes like glutathione (GSH), superoxide dismutase (SOD), glutathione peroxidase (GPx), and catalase. These mechanisms lead to the generation of reactive oxygen species, subsequently amplifying the function of NF-κB, a key mediator in the inflammatory response. Additionally, reactive oxygen species exert influence on the Nrf-2 pathway, which plays a critical role in diminishing the concentrations of reactive oxygen species. Additionally, cadmium upregulates metallothionein, a protein that binds to cadmium and facilitates its excretion. Where *A. lappa L*. prevents the production of reactive oxygen species by inhibiting the depletion of antioxidant enzymes. *A. lappa L*. also inhibits the inhibition of Nrf-2 and activation of NF-κB. *ETC* electron transport chain, *SOD* superoxide dismutase, *GPx* glutathione peroxidases, *GSH* glutathione, *NF-κB* nuclear factor kappa B, *Nrf 2* nuclear factor erythroid 2-related factor 2

Cadmium toxicity is a well-documented concern owing to its deleterious effects on various organ systems and its extensive dispersion in the environment. The hepatic tissue is particularly susceptible to distinctive pathological alterations induced by cadmium, even at relatively low doses over short or prolonged periods. The presence of cadmium in the human organism not only interferes with the normal physiological functions of cells and their subcellular structures but also hinders the absorption and processing of calcium. Binding to glutathione-stimulating hormone (GSH), cadmium leads to a reduction in GSH levels and the formation of protein chelators derived from GSH (Fig. 4).

The impact of cadmium can lead to either a decrease or an increase in the functions of diverse antioxidative enzymes. Metallothionein assumes a critical function in the toxicity caused by cadmium. Research has demonstrated that Metallothionein interacts with cadmium, altering its toxicity within cells [65]. When individuals experience acute exposure to cadmium, a protein like metallothionein that binds cadmium shields the testes from its toxic effects [66]. Metallothionein, comprising Metallothionein-1 and Metallothionein-2, is indispensable for regulating the levels of Zn and Cu and safeguarding against cadmium toxicity in various body tissues [67]. Furthermore, Metallothionein serves as an indicator of cadmium toxicity, with cadmium prompting their production following exposure [68]. Moreover, Metallothionein diminishes oxidative damage to DNA triggered by cadmium, underscoring its protective function against Cadmium-induced harm [69]. Moreover, cadmium induces a decline in ATP levels within cells, ultimately leading to the breakdown of the mitochondrial membrane. Consequently, there is an elevation in calcium and cytochrome C levels in the cell matrix, facilitating the activation of various caspases by attaching to apoptosis protease activating factor 1 (Apaf-1), thereby instigating apoptosis [70].

The root extract of *A. lappa L*. at a dosage of 300 mg/kg has demonstrated the presence of antioxidant compounds, which are responsible for its hepatoprotective properties against liver damage induced by cadmium. *A. lappa L*. root extract stimulates an augmentation in the number of nuclei per unit area and the number of binuclear hepatocytes, suggesting a process of tissue rejuvenation. The antioxidative capabilities of *A. lappa L*. root extract aid in neutralizing free radicals, diminishing the oxidative stress triggered by cadmium, and fostering the recuperation and restoration of liver cells harmed by cadmium [71]. *A. lappa L*. root extract inhibits the NF-κB responsible for inflammation and activates the Nrf-2 pathway responsible for reducing inflammation.

### Lead-induced toxicity

**Fig. 5:**
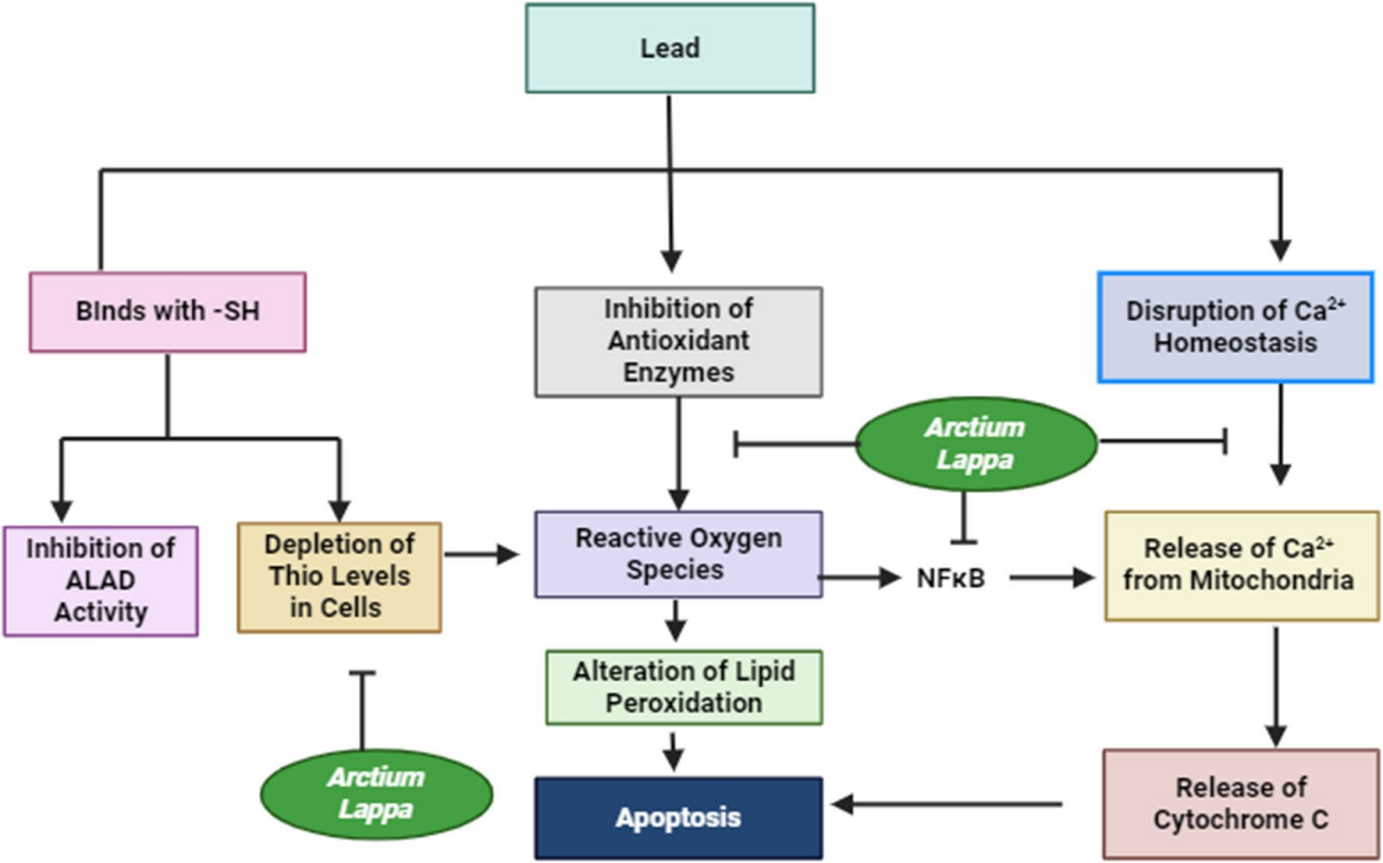
The schematic illustration depicts the mechanism by which lead induces toxicity. The illustration demonstrates that lead exerts its toxic effects by decreasing levels of crucial antioxidant enzymes (GSH, SOD, GPx, and catalase). This depletion results in the generation of reactive oxygen species and a reduction in thiol levels, as lead binds to sulfhydryl (-SH) groups. Therefore, an increase in NF-κB expression occurs, which is a crucial transcription factor involved in the enhancement of inflammatory responses. Lead also disrupts calcium (Ca^2+^) homeostasis, leading to the release of Ca^2+^ from mitochondria, which triggers the release of cytochrome C. The release of cytochrome C subsequently activates the caspase system, initiating apoptosis. Notably, NF-κB also facilitates the release of cytochrome C. Furthermore, lead inhibits the activity of ALAD, an enzyme crucial for the second step of heme biosynthesis. ALAD's dysfunction further disrupts calcium homeostasis. *NF-κB* nuclear factor kappa B, *ALAD* δ-aminolevulinic acid dehydrogenase

The element Lead (Pb) is recognized as a hazardous metal capable of eliciting a diverse array of behavioral, biochemical, and physiological responses in the human population [72]. The liver, being highly susceptible to the toxic effects of Pb, is prone to accumulation of this element. Pb exposure can lead to an increase in oxidative stress, which in turn can cause harm to the liver. The excessive presence of free radicals and the activation of inflammatory pathways can induce the death of hepatocytes. Moreover, the induction of oxidative stress by Pb has the potential to initiate the mitochondrial apoptotic pathway, leading to the liberation of cytochrome C, stimulation of caspase 3, and eventual cellular demise. Apart from oxidative stress, inflammation also contributes to the toxicity caused by Pb. Pro-inflammatory cytokines and reactive oxygen species can activate inflammatory pathways, thus exacerbating liver injury [73] (Fig. 5).

Research studies have demonstrated that the application of the extract derived from the root of *A. lappa L*. can protect against liver injury induced by lead (Pb). The oral delivery of *A. lappa L*. root extract was executed at a dose of 200 mg/kg over a consecutive period of seven days. This effect is achieved by mitigating oxidative stress and inflammatory reactions. The initiation of the Akt/GSK-3β signaling pathway is prompted by the introduction of *A. lappa L*. root extract. The activation of Akt initiates the phosphorylation and subsequent deactivation of GSK-3β, consequently hindering its participation in inflammatory processes and cell demise through apoptosis. This mechanism further amplifies the hepatoprotective properties of *A. lappa L*. root extract by diminishing oxidative harm and inflammation within the liver by heightening antioxidant defenses, impeding lipid peroxidation and the generation of nitric oxide, and augmenting the levels of cellular antioxidants like glutathione and superoxide dismutase in the livers of rats induced with lead [73].

### Chromium toxicity

**Fig. 6:**
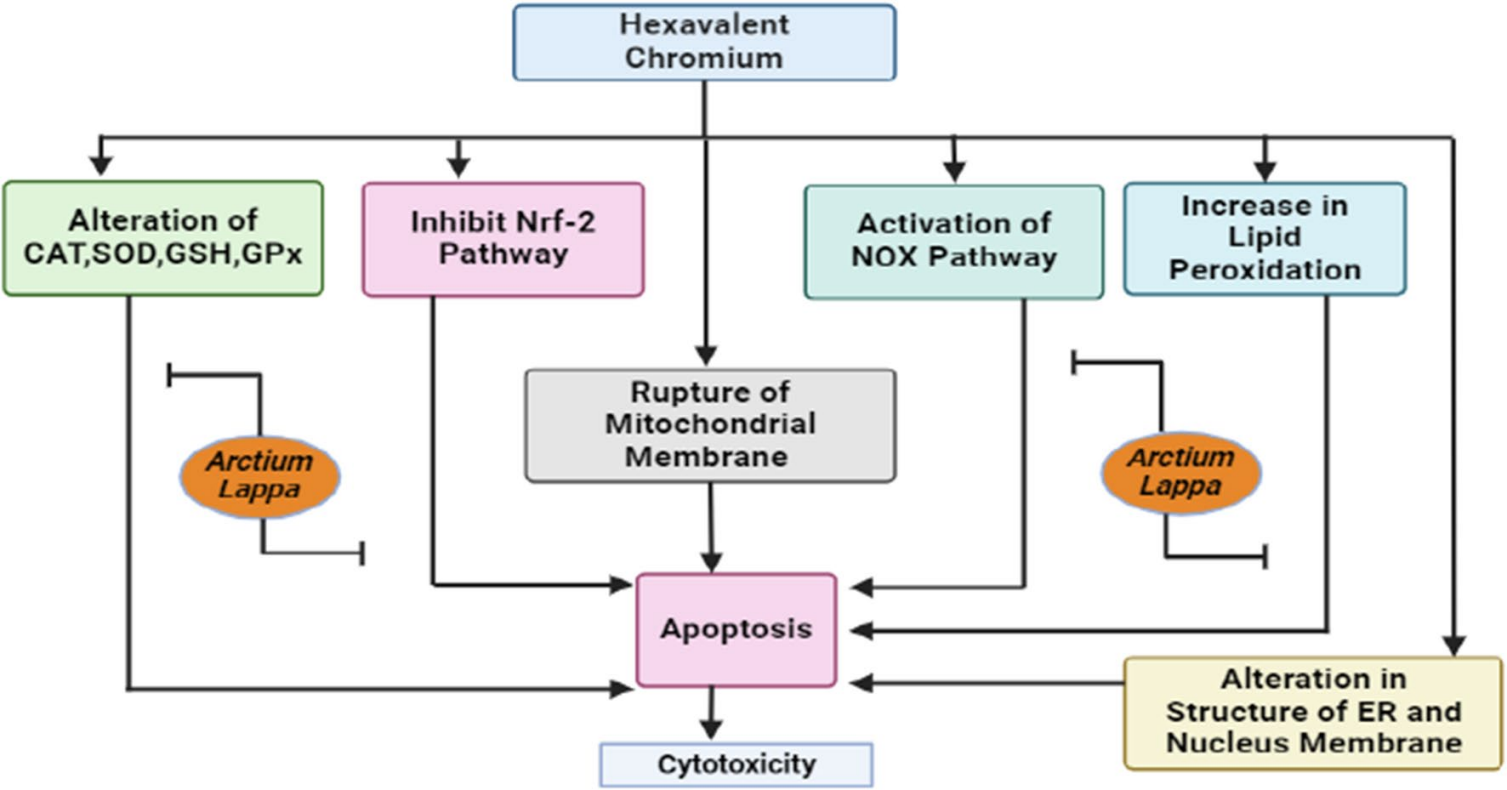
The schematic diagram illustrates the toxic effects caused by hexavalent chromium. It shows the mechanism through which chromium induces cytotoxicity by activating the NOX pathway, increasing lipid peroxidation, inhibiting the Nrf-2 pathway, causing mitochondrial membrane rupture, altering antioxidant enzymes (CAT, SOD, GSH, and GPX), and damaging the endoplasmic reticulum and nuclear membrane. These diverse pathways trigger inflammation and oxidative stress, ultimately leading to apoptosis and cytotoxicity. *A. lappa L*. inhibits the alteration in the antioxidant enzyme and prevents inhibition of the Nrf-2 pathway. *A. lappa L*. also shows its mechanism of action by inhibiting the lipid peroxidation and NOX pathway. *SOD* superoxide dismutase, *GPx* glutathione peroxidases, *GSH* glutathione, *NF-κB* Nuclear factor kappa B, *Nrf-2* nuclear factor erythroid 2-related factor 2, *NOX* NADPH oxidase, *ER* endoplasmic reticulum

Exposure to hexavalent chromium (Cr VI) leads to an escalation of levels of reactive oxygen species in the hepatic tissue of rats, zebrafish, and human liver cells, thus indicating the presence of oxidative stress. Subsequently, the dysregulation of the Nrf-2 pathway, which is associated with the antioxidant response, occurs post-chromium exposure, causing an imbalance in redox and disruption of the equilibrium of the hepatic antioxidant system. Moreover, changes in the acetylation and methylation of histones, as well as abnormalities in gene methylation, have been detected in the liver post-exposure to chromium. Furthermore, hepatocytes exposed to chromium display structural alterations at an ultrastructural level, such as an enlargement of the rough endoplasmic reticulum, modifications in the configuration of the mitochondrial and nuclear membranes, the occurrence of pycnotic nuclei, and the emergence of cytoplasmic vacuolization [74] (Fig. 6).

The oil extracted from *A. lappa L*. has been extensively researched for its potential capability to protect against oxidative stress induced by CrVI and gamma radiation. When administered intra-gastrically at a dosage of 2.5 ml/kg of body weight for a duration of 14 days, *A. lappa L*. oil demonstrates antioxidant properties, thereby aiding in the neutralization of reactive oxygen species generated by CrVI and gamma radiation. Studies have shown that *A. lappa L*. oil possesses the ability to regulate the oxidation processes of lipids and proteins, enhance the activity of antioxidant enzymes, and replenish the levels of endogenous antioxidants like glutathione. The antioxidative capacity of *A. lappa L*. oil plays a crucial role in alleviating oxidative damage to vital organs such as the liver, kidney, and blood cells, which may arise from the combined effects of CrVI and gamma radiation [75].

### *A. lappa L*. root extract in drug-induced toxicity

**Fig. 7:**
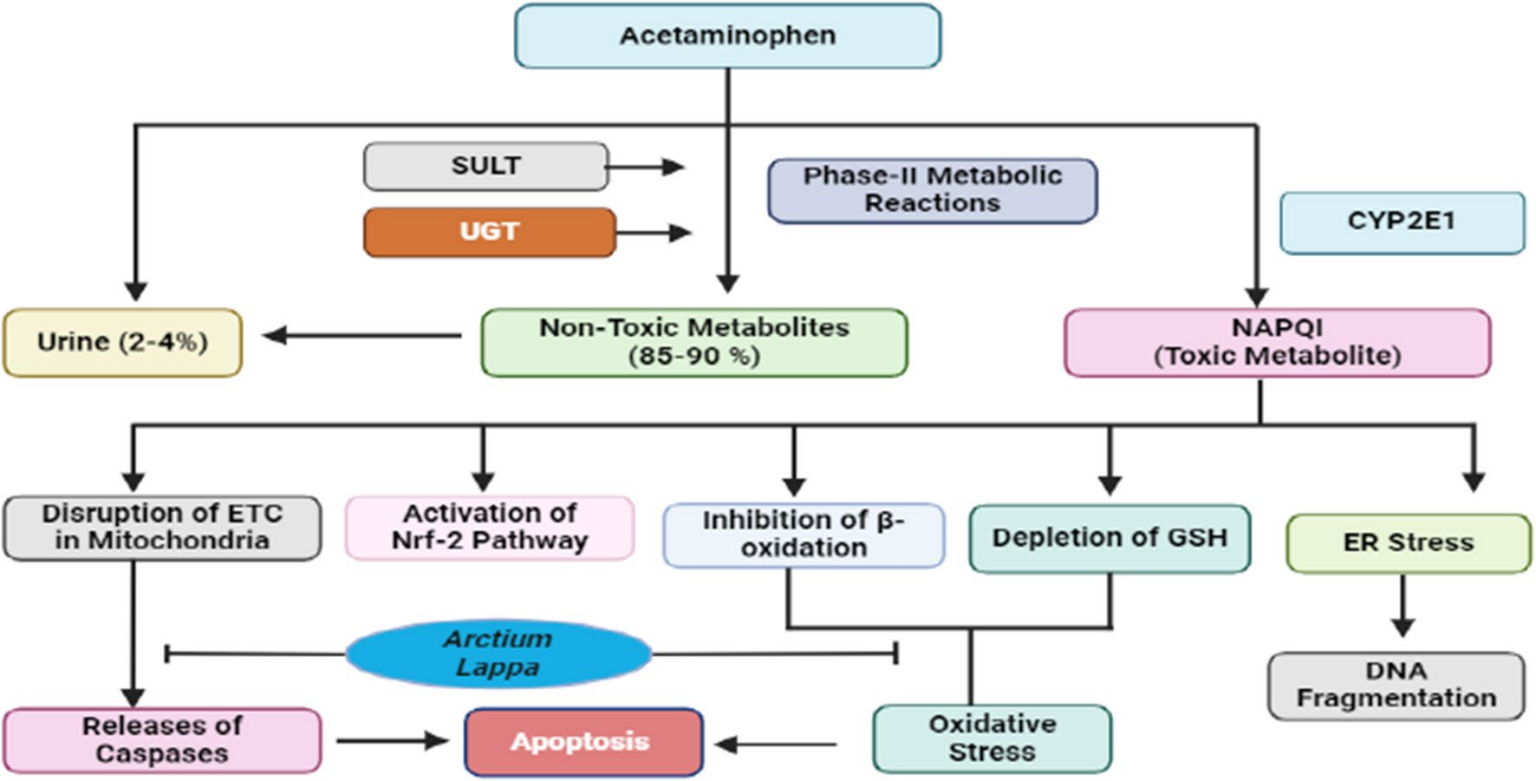
The diagrammatic representation elucidates the mechanism of toxicity of acetaminophen. Acetaminophen undergoes metabolism through various pathways. During the phase-II reaction, acetaminophen is converted into non-toxic metabolites by SULT and UGT, while the remainder is excreted in urine; CYP2E1 transforms it into the toxic metabolite NAPQI. NAPQI is accountable for the disruption of the Electron Transport Chain (ETC) in mitochondria, hindering β-oxidation and resulting in the accumulation of lipids in the liver. Furthermore, NAPQI binds to the –SH group, leading to a depletion of Glutathione (GSH). These processes culminate in the generation of oxidative stress, where the disruption of the ETC in mitochondria triggers the caspase system, ultimately resulting in apoptosis. Additionally, NAPQI induces Endoplasmic Reticulum (ER) stress, causing DNA fragmentation. *A. lappa L*. prevents the disruption of ETC and depletion of GSH and inhibits the inhibition of β-oxidation. *GSH* glutathione, *ETC* electron transport chain, *UGT* glucuronosyltransferases, *SULT* sulfotransferases, *NAPQI* *N*-acetyl-*p*-benzoquinone imine, *Nrf-2* nuclear factor erythroid 2-related factor 2, *ER* endoplasmic reticulum

Acetaminophen toxicity represents a prevalent occurrence of drug-induced side effects on a global scale, which can result in substantial harm to the liver and the development of hepatotoxicity. The phenomenon of acetaminophen-induced hepatotoxicity is widely recognized and can bring about liver injury and failure. The United States Food and Drug Administration advises the use of N-acetyl cysteine, a recognized antioxidant, as the sole therapeutic choice for patients who have overdosed on acetaminophen [76]. The toxic nature of acetaminophen is facilitated by the generation of a reactive metabolite known as N-acetyl-p-benzoquinone imine (NAPQI), which causes a depletion of glutathione and forms covalent bonds with proteins [77, 78]. This covalent attachment and glutathione depletion are interconnected with the emergence of hepatic necrosis [79]. The manifestation of acetaminophen-induced hepatotoxicity is characterized by changes in histopathology, including hemorrhaging, congestion, hepatocyte necrosis, and lipid peroxidation [80] (Fig. 7)

The administration of *A. lappa L*. root extract at a dosage of 300 mg/kg orally demonstrated beneficial effects on acetaminophen-induced hepatotoxicity in rats. Utilization of *A. lappa L*. root extract led to a decrease in the levels of alanine transaminase, aspartate aminotransferase, and alkaline phosphatase in the group injected with acetaminophen. Additionally, the root extract of *A. lappa L*. hindered the presence of malondialdehyde, a marker of oxidative stress, in the group treated with acetaminophen. Evaluation through histopathologic examination unveiled that treatment with *A. lappa L*. root extract mitigated hepatic cell necrosis, lymphocyte infiltration, and vacuolation linked to acetaminophen-induced hepatotoxicity [81].

## Pharmacological effect of *A. lappa L*. root extract

**Fig. 8:**
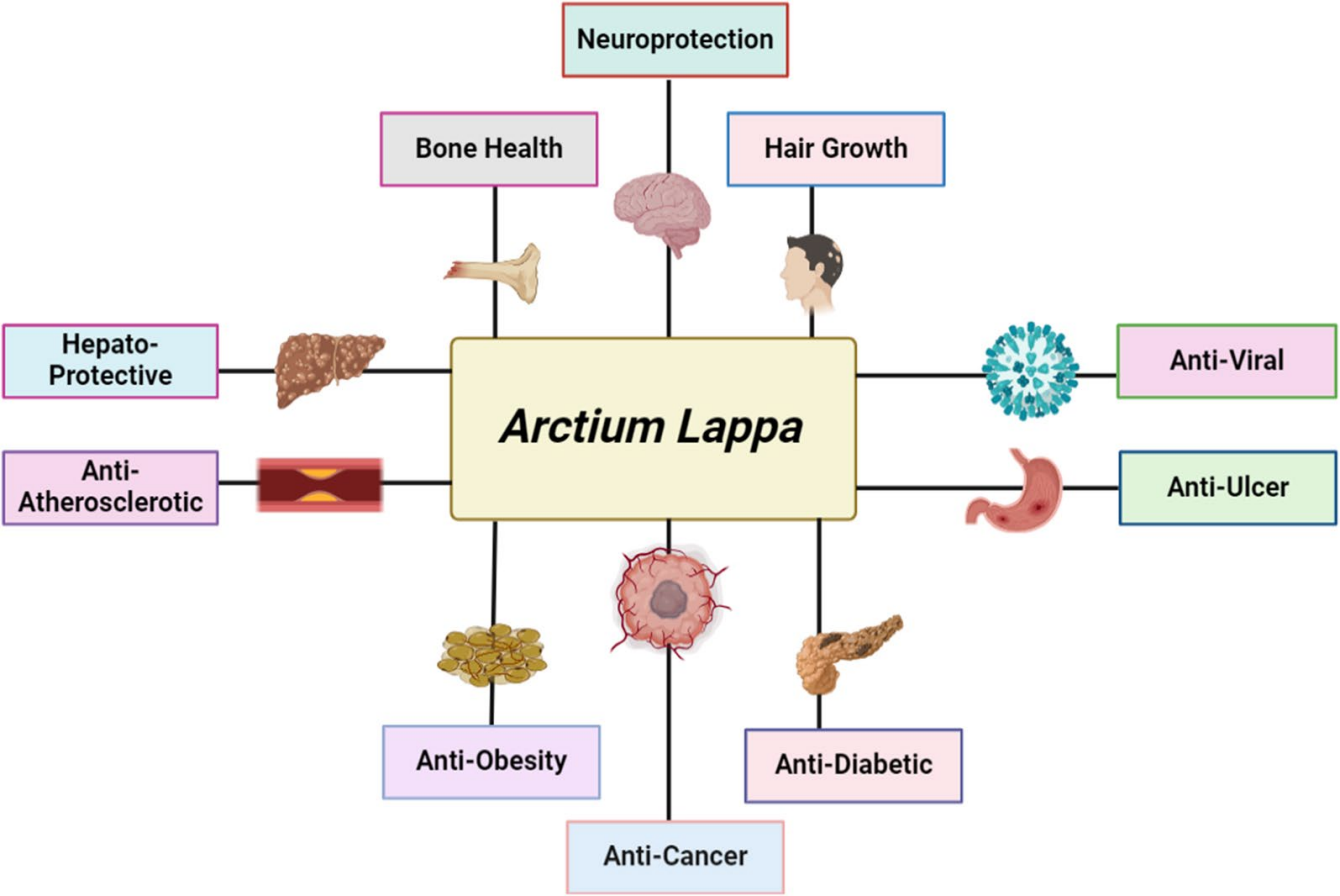
Schematic representation of therapeutic actions of *A. lappa L*. root extract

*A. lappa L*. has been found to have various pharmacological effects. It has been shown to have antiviral, anti-inflammatory, hypolipidemic, and antidiabetic effects [82]. The root of *A. lappa L*. exhibits antioxidant characteristics attributed to the existence of chlorogenic acid. [83]. The utilization of this substance has a historical presence in the field of Chinese medicine and has demonstrated properties related to promoting bile secretion, enhancing liver function, boosting the immune system, and reducing inflammation. Studies have been conducted on the root of *A. lappa L*. to explore its potential therapeutic advantages in addressing conditions such as non-alcoholic fatty liver disease, preventing diverticulitis, managing diabetic nephropathy, combating photoaging, treating acne, and alleviating inflammation [84, 85]. It has also been found to have diuretic properties and may be beneficial for rheumatism, gastritis, gout, throat pain, and arthritis as shown in Fig. 8.

The root is composed of bioactive compounds such as fructo-oligosaccharides and flavonoids, which play a significant role in its pharmacological characteristics. Overall, *A. lappa L*. shows promise as a natural remedy with various health benefits.

### Anti-diabetic

Millions of people worldwide suffer from diabetes. Diabetes mellitus has the potential to cause both acute and chronic systemic complications. It is widely recognized as a significant public health concern in the twenty-first century, characterized by an increasing prevalence and potentially devastating consequences [86]. Diabetes mellitus affects numerous organs and tissues, including but not limited to the pancreas, liver, kidney, nerves, blood vessels, retina, and sperm quality [87, 88]. The global burden of diabetes has experienced a substantial rise over the past 28 years, albeit with variations in different regions and countries [89]. This condition is intricately linked with macrovascular and microvascular complications, including coronary heart disease, strokes, peripheral arterial disease, diabetic kidney disease, retinopathy, peripheral neuropathy, and heart failure [90]. For centuries, herbal medicine has been employed in the management of diabetes mellitus. Herbal remedies utilize the medicinal properties of botanicals and extracts derived from plants to modulate levels of glucose and manage the manifestations linked to diabetes mellitus [91, 92]. Numerous botanicals and botanical extracts, such as garlic, neem, coriander, ivy gourd, papaya, jamun, tulsi, and aloe vera, have traditionally been employed in herbal therapies for diabetes. Research studies have demonstrated that herbal nutritional supplements can effectively diminish blood glucose levels, HbA1c levels, and urea levels in individuals with diabetes mellitus [93, 94].

**Fig. 9:**
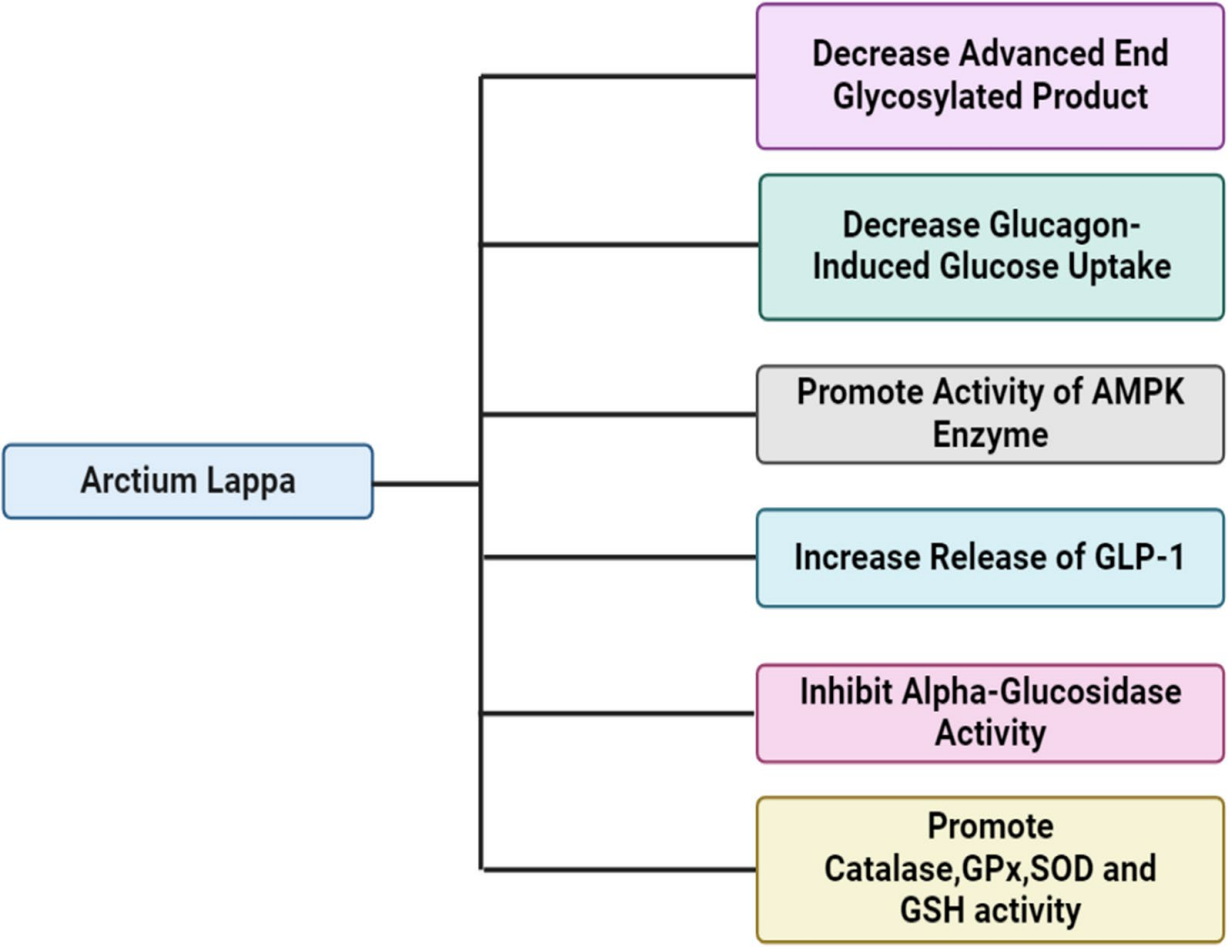
The schematic diagram depicts the mechanisms through which *A. lappa L*. root extract demonstrates its efficacy in diabetes mellitus. *A. lappa L*. root extract reduces the presence of advanced end glycosylated products, which are a group of altered proteins or lipids formed when sugars like glucose and fructose bind to them, disrupting cellular processes and increasing inflammation. Furthermore, it inhibits glucagon-induced glucose uptake, leading to elevated blood sugar levels, and hinders the activity of the enzyme alpha-glucosidase, responsible for breaking down complex carbohydrates into simpler forms. Furthermore, the extract amplifies the role of the AMPK enzyme, crucial for the regulation of triglyceride synthesis, sterol synthesis, and glucose uptake. Additionally, it enhances the effectiveness of GLP-1 in promoting insulin secretion, diminishing glucagon secretion, and prolonging gastric emptying. Additionally, it also stimulates various antioxidant enzymes to thwart lipid peroxidation and enhance mitochondrial function. *AMPK* AMP-activated protein kinase, *GLP-1* glucagon-like peptide-1, *GSH* glutathione, *SOD* superoxide dismutase, *GPx* glutathione peroxidases

*A. lappa L*. has been the subject of scientific investigation concerning its potential therapeutic properties in the context of diabetes mellitus. Multiple scholarly inquiries have demonstrated that *A. lappa L*. root extract exhibits anti-diabetic characteristics, encompassing enhancements in glucose homeostasis and mitigation of insulin resistance [95]. *A. lappa L*. root extract primarily exerts its effects by employing various mechanisms to regulate type-2 diabetes and mitigate the associated complications of diabetes mellitus that are explained in Fig. 9.

#### Adenosine monophosphate-activated protein kinase (AMPK) pathway

The AMPK pathway plays a pivotal role in the development of diabetes mellitus. AMPK, an enzymatic molecule, acts as a regulatory switch for metabolic processes by sensing the levels of AMP and the absence of glucose within cells. The activation of AMPK triggers energy-producing metabolic pathways, while simultaneously inhibiting energy-consuming pathways and cellular activities. In the context of diabetes, a malfunctioning of AMPK activity has been observed, and drugs such as metformin work by modulating the activity of AMPK. The activation of AMPK promotes the uptake of glucose into cells and suppresses the production of glucose within cells. Moreover, AMPK is accountable for upholding mitochondrial stability and speeding up autophagy, both of which contribute to the emergence of insulin resistance. Consequently, the stimulation of AMPK is viewed as a promising therapeutic strategy for the control of diabetes mellitus and its related complications. Various natural substances such as berberine, quercetin, and resveratrol have exhibited potential in modulating and activating the AMPK pathway, thereby offering alternative approaches for treating diabetes [96–98].

**Fig. 10:**
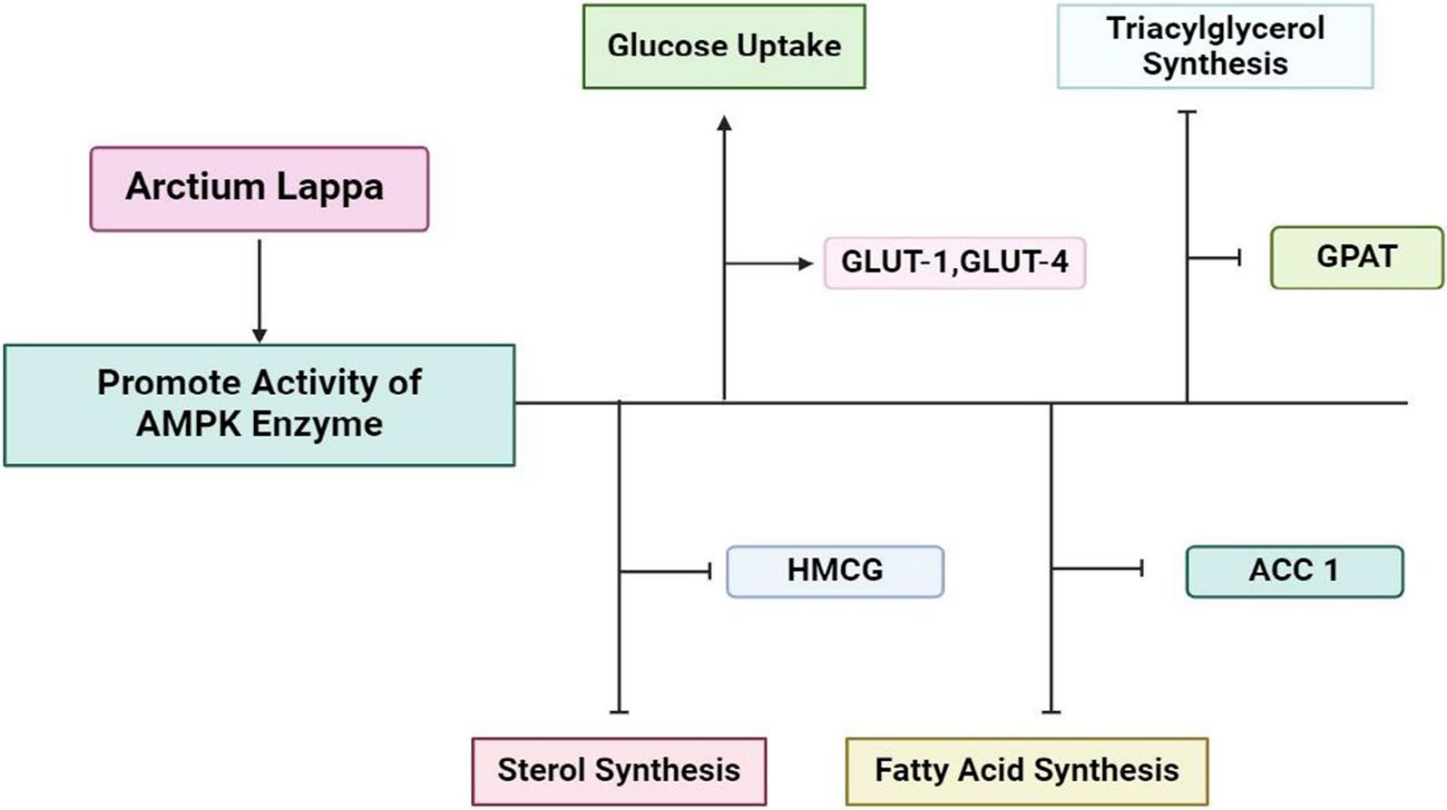
The schematic diagram illustrates the processes through which the activation of the AMPK enzyme is facilitated by *A. lappa L*. Furthermore, the AMPK enzyme enhances glucose uptake by stimulating GLUT-1 and GLUT-4, particularly in skeletal muscle and adipose tissue. The stimulation of AMPK activity inhibits sterol synthesis, triglyceride synthesis, and fatty acid synthesis by suppressing the HMCG, ACC1, and GPAT enzymes, respectively. *AMPK* AMP-activated protein kinase, *GLUT *glucose transporter, *ACC1* acetyl-CoA carboxylase 1, *HMCG* 3-hydroxy-3-methylglutaryl-CoA reductase, *GPAT* glycerol-3-phosphate acyltransferase

Arctigenin a component of *A. lappa L*. extract elicits the activation of AMPK and ameliorates glucose and lipid metabolism in diabetes mellitus [99–101]. It enhances AMPK phosphorylation and stimulates glucose uptake in myocytes while inhibiting hepatic gluconeogenesis and lipogenesis. AMPK phosphorylation is improved by it, and it promotes the uptake of glucose in myocytes, while also hindering hepatic gluconeogenesis and lipogenesis. Additionally, it enhances the creation of mitochondria and the oxidation of fatty acids in muscle tissues. The activation of AMPK by Arctigenin is brought about through calmodulin-dependent protein kinase kinase and serine/threonine kinase 11-dependent pathways (Fig. 10).

Furthermore, it impedes respiratory complex I, leading to the activation of AMPK. The administration of arctigenin contributes to the improvement of glucose and insulin tolerance, reduction of inflammation, and enhancement of productive outcomes in gestational diabetes mellitus. Additionally, arctigenin inhibits Th17 cells and activates AMPK, thus exhibiting anti-inflammatory and immunosuppressive effects The findings indicate the possible therapeutic application of arctigenin as a pharmacological intervention for ailments like multiple sclerosis and various autoimmune inflammatory conditions [102].

#### Glucagon-induced glucose output

Glucagon has a biphasic effect on hepatic glucose production. Initially, it stimulates a rapid, transient peak in hepatic glucose production, followed by a slow reduction in hepatic glucose production to a rate of 1.5-fold basal [103]. Examination of a desiccated *A. lappa L*. root extract showed the presence of a majority of caffeoylquinic acid and its derivatives, among which 1,5-di-*O*-caffeoyl-4-*O*-maloylquinic acid was identified as the most predominant compound [104]. The root extract of *A. lappa L*. demonstrated an ability to increase glucose uptake in cultured L6 myocytes and decrease glucagon-induced glucose production from isolated rat hepatocytes, suggesting its potential as a treatment for hyperglycemia. In vivo studies demonstrated that the *A. lappa L*. root extract improved glucose tolerance following both intraperitoneal and oral sub-chronic administration [105]. A separate investigation confirmed that the ethanolic extract of *A. lappa L*. root extract significantly lowered blood glucose levels and increased insulin levels in diabetic rats [12]. Additionally, the extract improved glucose tolerance in normoglycemic rats. Moreover, the extract decreased serum total cholesterol, triglycerides, and low-density lipoprotein levels, while raising high-density lipoprotein levels [106].

#### Alpha-glucosidase activity

Alpha-glucosidase inhibitors exhibit the ability to prolong the process of absorbing consumed carbohydrates, thereby diminishing the levels of glucose in the blood after a meal [107]. The methanol extract of *A. lappa L*. root exhibited inhibitory activity against α-glucosidase, with the compound sitosterol-β-d-glucopyranoside being identified as the main inhibitory compound [108].

#### Glucagon-like peptide

GLP-1, also known as glucagon-like peptide, is a peptide hormone like insulin that is produced by L-cells located in the epithelium of the gastrointestinal tract. Its functions include the stimulation of insulin release, regulation of hunger, and various impacts on the management of blood sugar levels and body weight. Pharmaceuticals like liraglutide, and dulaglutide, which are agonists of the GLP-1 receptor, have been created to address type 2 diabetes and obesity. These agonists not only enhance the control of blood sugar levels and promote weight reduction but also offer protection against heart disease [108, 109]. *A. lappa L*. has exhibited the ability to enhance the release of GLP-1 in animal models. The introduction of *A. lappa L*. root extracts, which are abundant in caffeoylquinic acid derivatives, led to an augmentation in glucose uptake and GLP-1 release in L6 myocytes and hepatic cell lines derived from rats. The amplified release of GLP-1 observed in animal models could potentially be ascribed to the interaction between specific compounds present in *A. lappa L*. root extract and bitter taste receptors in the intestines, thereby resulting in an escalated release of incretins, including GLP-1 [110].

#### Antioxidant activity

The pathogenesis of type 2 diabetes and its complications are heavily influenced by oxidative stress. Individuals diagnosed with type 2 diabetes exhibit elevated levels of oxidative stress markers, including lipid peroxidation, protein carbonyls, and hydrogen peroxide production [111, 112]. These markers are indicative of an imbalance in antioxidant defense mechanisms, with alterations observed in the activity of antioxidant enzymes. Moreover, the modulation of crucial factors responsible for maintaining equilibrium in oxygen and metabolic processes, including HIF-1α and mTOR, is similarly influenced in individuals with type 2 diabetes. Additionally, the excessive intake of dietary fats and impaired mitochondrial function exacerbates the progression of oxidative stress and insulin resistance in patients diagnosed with type 2 diabetes [113–115]. Burdock root extract prevents the depletion of antioxidant enzymes and decreases oxidative stress. Moreover, burdock root extract has been reported to contain active compounds that may have effects on mTOR receptor signaling pathways [116].

#### Advanced end glycosylated products (AGE)

AGEs are produced through the reaction between sugars and proteins or lipids, a process known as glycation. In individuals with diabetes, there is an elevated production and accumulation of AGEs due to elevated blood sugar levels. These AGEs can result in inflammation, oxidative stress, and tissue damage, thereby causing dysfunction in various organs and systems within the body. Moreover, they can impede the proper functioning of insulin-producing cells in the pancreas, thereby exacerbating blood sugar regulation. Furthermore, AGEs can contribute to insulin resistance, thereby hindering the ability of cells to respond to insulin and regulate blood sugar levels. The consumption of burdock root, which contains fructo-oligosaccharides, has been found to exhibit antidiabetic effects. Such fructo-oligosaccharides aid in the regulation of blood glucose levels by promoting the growth of beneficial gut bacteria, which consequently enhances glucose metabolism. Additionally, *A. lappa L*. root extract is rich in chlorogenic acid, an antioxidant that lowers the presence of AGEs and thus reduces oxidative stress [117].

### Cancer

*A. lappa L*. root extracts have been found effective in various cancers and act on multiple pathways. Root extracts of *A. lappa L*. have been utilized in experimental studies involving different types of human cancer cell lines such as HeLa, MCF-7, and Jurkat T cells. The analysis revealed that the extracts displayed considerable efficacy in combating cancer in these cell lines, inducing apoptosis and instigating alterations in morphology. The ethyl acetate extract derived from the root of *A. lappa L*. has displayed considerable potential in the fight against cancer by triggering intrinsic apoptosis via the perturbation of mitochondrial membrane potential and the initiation of caspase 3/7 activation [118]. *A. lappa L*. root extract has also exhibited robust activity against breast cancer cells (MCF-7) and normal cells, suggesting its potential as an agent for combating cancer. *A. lappa L*. root extracts, especially the *n*-hexane fraction, have demonstrated proapoptotic effects on breast cancer cells (MCF-7) through the upregulation of p53, TGF-β, and NF-κB signaling pathways, leading to cellular demise. The key bioactive components in the *n*-hexane fraction, specifically stigmasterol, s-sitosterol, and 3-*O*-acetyl lupeol, significantly contribute to the anti-cancer attributes of this fraction by hindering the metastatic progression of breast cancer cells. This mechanism involves the inhibition of cell proliferation, migration, invasion, and colonization [119]. *A. lappa L*. root extract has been studied for its potential to address colon cancer. It is conceivable that *A. lappa L*. root extract could potentially exert inhibitory effects on cellular proliferation by modulating signaling pathways related to cell cycle regulation, apoptosis, or DNA repair. Further investigation is necessary to clarify the precise molecular pathways and targets involved in the inhibitory effects of *A. lappa L*. on cell proliferation [120].

The extract derived from the *A. lappa L*. demonstrates potential as a beneficial treatment for non-alcoholic steatohepatitis, thereby potentially lowering the risk of hepatocellular carcinoma formation. The observation of significant concentrations of chlorogenic and caffeic acids in the root extract of *A. lappa L*. was documented, resulting in a decline in overall levels of fatty acid and lipid hydroperoxide following the administration of the extract. Moreover, there was an enhancement in the hepatic activities of the antioxidant enzymes superoxide dismutase and catalase. In addition, the application of burdock extract led to a decrease in the size of GST-P + remodeling preneoplastic lesions and showed a tendency to reduce hepatocyte proliferation (Ki-67) within these lesions. These findings indicate that brief exposure to *A. lappa L*. extract could alleviate the progression of remodeling preneoplastic lesions in hepatocarcinogenesis linked to non-alcoholic steatohepatitis [121].

### Atherosclerosis

Atherosclerosis represents a persistent inflammatory state impacting the arterial wall, characterized by the development of plaques consisting of lipids, connective tissue, and immune cells within the arteries. It serves as the primary determinant of cardiovascular disease, the leading cause of death worldwide [122]. The advancement of atherosclerosis unfolds through specific phases, initiating with endothelial dysfunction and concluding with plaque rupture [123]. Various risk factors, such as dyslipidemia, hypertension, diabetes mellitus, smoking, elevated homocysteine, and hormonal imbalances, have been associated with the progression of the condition [124]. Root extracts from *A. lappa L*. demonstrated protective effects against atherosclerosis induced by a high-fat diet in quails by enhancing lipid profile and antioxidant status, conceivably through hypolipidemic and antioxidant mechanisms. Quails (Coturnix coturnix) were subjected to a high-fat diet, either with *A. lappa L*. or the positive control simvastatin. Blood samples were collected pre-treatment, after 4.5 weeks, or 10 weeks for lipid profile assessment. Results indicated that the high-fat diet significantly deteriorated lipid profile and antioxidant status in quail serum, while *A. lappa L*. extract effectively reversed these changes like simvastatin [125]. Another study demonstrated that both in vivo and in vitro investigations showed that treatment with *A. lappa L*. leaves substantially reduced TC and LDL-C, increased HDL-C, and reduced cholesterol accumulation in the liver and aorta of the rat model and the foam cell model. Importantly, outcomes from both in vivo and in vitro experiments suggested that *A. lappa L*. leaves regulate the PPARG/LXRα and AMPK/SIRT1 signaling pathways [126].

### Anti-allergic

Numerous investigations have been conducted to explore the anti-allergic response of *A. lappa L*. The butanol extract derived from *A. lappa L*. impedes the secretion of β-hexosaminidase in RBL-2H3 cells stimulated by antigens, thus showcasing its ability to hinder mast cell degranulation. Administration of the butanol extract from *A. lappa L*. also dampens the production of interleukin-4 and interleukin-5 in primary splenocytes exposed to Concanavalin A, indicating its potential to alleviate allergic inflammation. Furthermore, the butanol extract of *A. lappa L*. diminishes the activation of mitogen-activated protein kinase and NF-κB in primary splenocytes treated with Concanavalin A, both of which are pivotal in the inflammatory process [127]. Another research study has demonstrated that the fruit extract and fermented version of *A. lappa L*. display anti-allergic properties by blocking the activation of the high-affinity IgE receptor triggered by the antigen-IgE complex. The fermented derivatives originating from the fruit extract of *A. lappa L*. have been shown to impede the phosphorylation of various proteins such as Lyn, Fyn, Syk, phosphoinositide phospholipase C (PLC)γ1/2, protein kinase C (PKC)δ, extracellular signal-regulated kinase 1/2 (ERK1/2), c-Jun N-terminal kinase 1/2 (JNK), p38, and Akt. These proteins play crucial roles in the FceRI signaling pathway and the expression of cytokines [128]. The primary bioactive component accountable for the effectiveness against allergies is arctigenin, which additionally inhibits IgE-mediated passive cutaneous anaphylaxis and the induction of anaphylactic shock by compound 48/80. Compound 48/80 is acknowledged for its role in initiating histamine discharge and is frequently employed in biochemical research to provoke mast cell degranulation [129].

### Neuroprotection

**Fig. 11:**
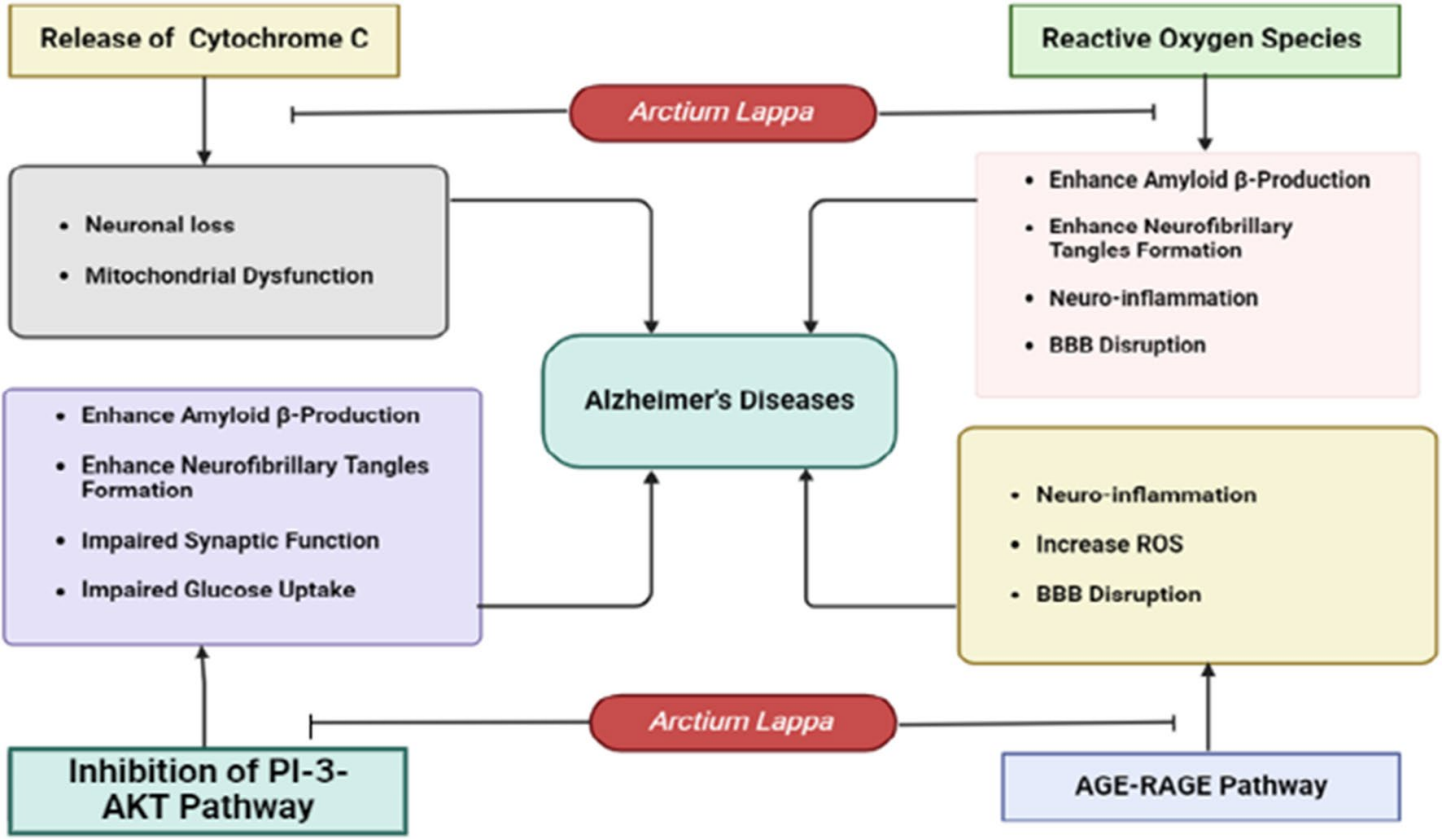
The schematic diagram depicts the mechanisms by which *A. lappa L*. operates in Alzheimer's disease. It enhances the PI-3k-Akt pathway by averting its inhibition, thereby reducing the production of amyloid-β, neurofibrillary tangles, impaired synaptic function, and impaired glucose uptake. Additionally, *A. lappa L*. mitigates the release of cytochrome C, thus preventing neuronal loss and mitochondrial dysfunction. Furthermore, it hinders the formation of free radicals, leading to the prevention of neuro-inflammation, blood–brain barrier disruption, and the formation of neurofibrillary tangles. *A. lappa L*. also inhibits the AGE-Rage pathway, which is accountable for neuro-inflammation and blood–brain barrier disruption. *BBB* blood–brain barrier, *ROS* reacting oxygen species, *AGE-RAGE* receptors for advanced glycation end products

The protective effects of *A. lappa L*. root extract against hydrogen peroxide-induced cellular damage in SH-SY5Y neuroblastoma cells have been demonstrated. Pre-treatment with *A. lappa L*. roots attenuated the cytotoxic effects of hydrogen peroxide, reversed nuclear condensation, and mitigated apoptosis. Furthermore, *A. lappa L*. roots enhanced the activity of antioxidant enzymes (such as GSH, GPx, and SOD), reduced levels of lipid peroxidation (MDA), inhibited the formation of reactive oxygen species (ROS), and preserved the mitochondrial membrane potential. Additionally, *A. lappa L*. roots increased the Bcl-2/Bax ratio, decreased the release of cytochrome *C*, and downregulated the functions and expressions of caspase-3 and caspase-9, thereby inhibiting apoptosis induced by hydrogen peroxide [130]. Furthermore, *A. lappa L*. roots diminished intracellular ROS levels and boosted the mitochondrial membrane potential in hydrogen peroxide-treated cells. *A. lappa L*. is recognized for activating the PI-3k-Akt signaling pathway while impeding the AGE-RAGE pathways and decreasing cytochrome C liberation (Fig. 11).

The antioxidative capabilities exhibited by burdock roots in safeguarding PC12 cells from oxidative stress, in conjunction with the discovery of quinic acid as a robust antioxidant agent, hold the potential to offer a favorable dietary antioxidant solution for individuals with Alzheimer's disease [131].

The investigation assessed the protective properties of the ethanol extract derived from burdock roots against oxidative stress in PC12 cells, utilizing the 2ʹ,7ʹ-dichlorofluorescein diacetate and 3-(4,5-dimethylthiazol-2-yl)-2,5-diphenyltetrazolium bromide assays. Oxidative stress has been associated with cognitive impairments triggered by Beta-amyloid1-42 in murine models. The application of burdock root ethanol extract exhibited improvements in working and reference memory in mice, as demonstrated by the Y-maze and passive avoidance evaluations. Levels of Malondialdehyde were quantified to evaluate lipid peroxidation. These findings strongly indicate that burdock root extract showcases neuroprotective properties in the context of neurodegenerative disorders such as Alzheimer's disease [132].

### Anti-obesity

**Fig. 12:**
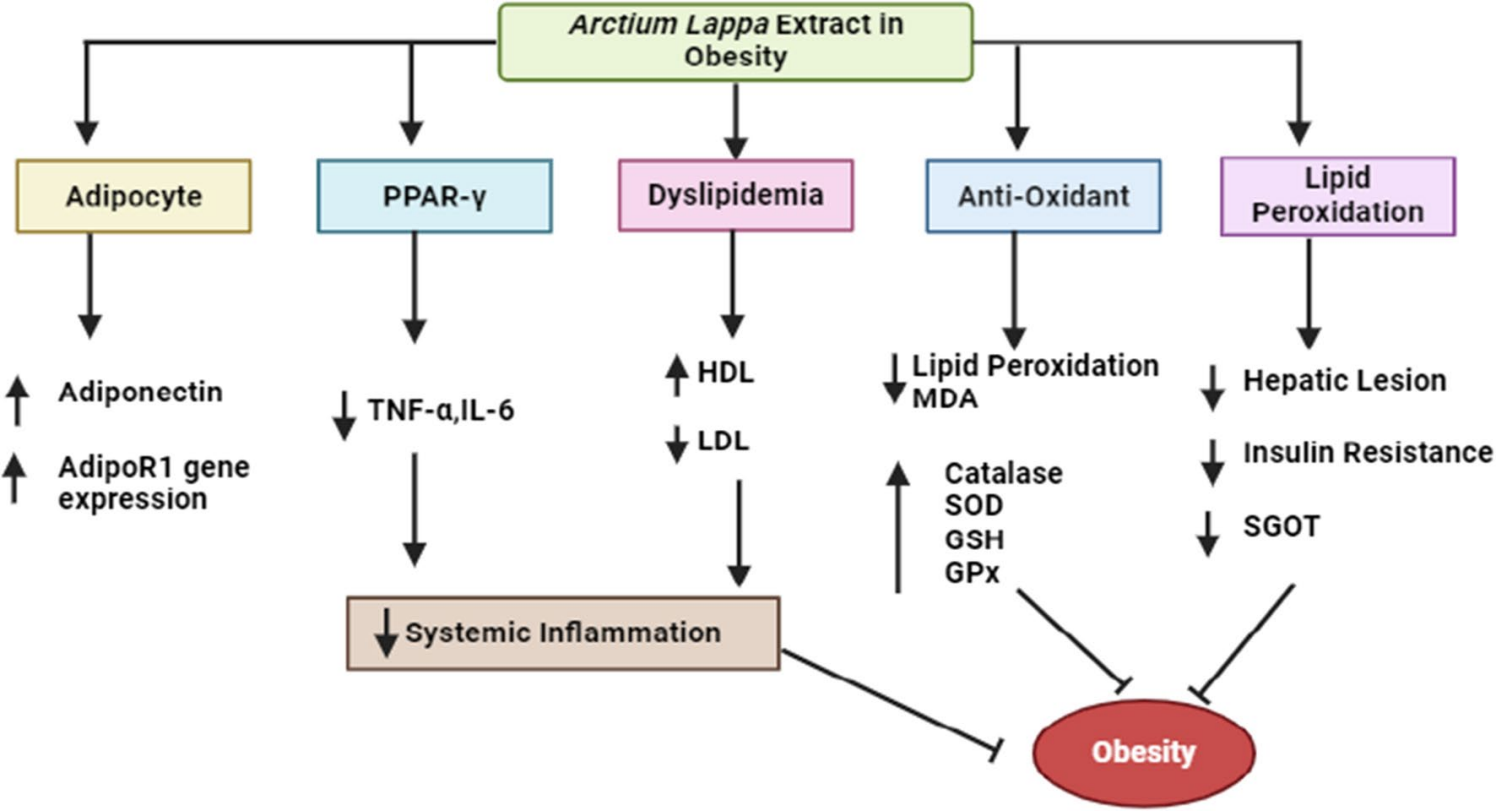
The schematic diagram of *A. lappa L*. extract impacts adipocytes and enhances adiponectin levels, thus improving insulin sensitivity, regulating fat metabolism, and exerting anti-inflammatory properties. *A. lappa L*. extract upregulates the expression of the AdipoR1 gene, which plays a crucial role in mediating the positive effects of adiponectin on fat metabolism and insulin sensitivity. Furthermore, *A. lappa L*. extract activates PPAR-γ, leading to increased insulin sensitivity and anti-inflammatory effects by reducing TNF-α and IL-6 levels. This extract also raises HDL cholesterol levels while lowering LDL cholesterol levels. Additionally, *A. lappa L*. extract mitigates oxidative stress by stimulating antioxidant enzymes and reducing lipid peroxidation, thus aiding in the improvement of hepatic lesions, and reducing insulin resistance. These various pathways collectively contribute to the treatment of obesity. *PPAR-γ* peroxisome proliferator-activated receptor gamma, *HDL* high-density lipoprotein, *LDL* low-density lipoprotein, *MDA* malondialdehyde, *SOD* superoxide dismutase, *GPx* glutathione peroxidases, *GSH* glutathione, *SGOT* serum glutamic-oxaloacetic transaminase

Obesity is characterized by chronic low-level inflammation, with *A. lappa L*. roots possessing anti-inflammatory properties. Prolonged intake of *A. lappa L*. roots may lead to a decrease in body weight. Research has shown the effectiveness of *A. lappa L*. root powder in reducing intestinal inflammation in obese rats fed a high-fat and high-sugar diet. Through modulation of the TLR4/NF-κB signaling pathway, *A. lappa L*. root extract powder influences immune and inflammatory responses, potentially contributing to its anti-obesity properties [133]. *A. lappa L*. root extract has demonstrated improvements in lipid profile parameters such as total cholesterol, low-density cholesterol, high-density cholesterol, triglycerides, and very-low-density cholesterol which are typically disrupted in cases of hyperlipidemia. Moreover, the extract exerts positive effects by lowering oxidative stress, as indicated by a decrease in thiobarbituric acid reactive substances and an increase in glutathione (GSH) and total thiol levels [134] (Fig. 12).

In another research study, the potential anti-obesity impacts of *A. lappa L*. on mice afflicted with diet-induced obesity and on cultured white and primary brown adipocytes are investigated. The results provide validation for the concept that *A. lappa L*. inhibits weight gain and decreases the size of white adipose tissue in obese mice, suggesting its potential as an effective therapeutic intervention for the treatment or prevention of obesity. The stimulation of AMP-activated protein kinase by the ethanol extract derived from *A. lappa L*. highlights its capacity to modulate metabolism and sustain energy equilibrium. Additionally, the ethanol extract of *A. lappa L*. inhibits the activity of PPAR-γ and CCAAT/enhancer-binding protein alpha (C/EBPα) in 3T3-L1 cells, which are genes linked to adipocyte differentiation. Furthermore, it enhances the activity of uncoupling protein 1 and peroxisome proliferator-activated receptor gamma coactivator 1-alpha (PGC-1α), genes recognized for their specificity to brown adipocytes, in primary cultured brown adipocytes [135].

### Rheumatoid arthritis

Research indicates that the extract derived from *A. lappa L*. can mitigate the severity of adjuvant arthritis in rodents by diminishing inflammation and safeguarding against joint degeneration. Osteoarthritis represents the prevailing age-related joint affliction distinguished by the deterioration of articular cartilage, resulting in discomfort, rigidity, and swelling in the impacted joints [136]. Predominantly, osteoarthritis affects the knee joint. The therapeutic mechanisms of *A. lappa L*. tea could involve its anti-inflammatory and antioxidant attributes. It has been hypothesized that this tea might lower the levels of inflammatory indicators such as high sensitivity to C-reactive protein and interleukin-6 (IL-6). Moreover, the consumption of *A. lappa L*. tea could augment the abundance of antioxidants like total antioxidant enzymes (GSH, GPx, and SOD). These antioxidative properties might aid in diminishing oxidative stress, a contributing factor to the advancement of osteoarthritis [137]. Burdock roots possess additional chemical constituents such as arctigenin, a distinguished bioactive element present in *A. lappa L*. This specific component exhibits potent anti-inflammatory characteristics and holds promise as a therapeutic remedy for acute inflammation as well as chronic conditions. Additionally, lapachol, another naturally occurring compound identified in *A. lappa L*., has been recognized as a prospective inhibitor of dihydroorotate dehydrogenase, showcasing immunosuppressive attributes. Consequently, it demonstrates potential in the treatment of rheumatoid arthritis [138].

### Skin disorders

The bioactive constituents existing in various components of burdock, such as the root, have been discovered to enhance the quality and texture of the skin, purify the bloodstream, and exhibit antioxidant characteristics [99]. The anti-acne attributes of the distinct peptide fraction derived from the *A. lappa L.* have been substantiated through initial biological investigations. The peptides isolated from *A. lappa L.* have also shown no toxicity to fibroblasts, indicating their potential safety for use. *A. lappa L.* contains low molecular weight peptides that have been found to exhibit anti-bacterial activity against gram-positive acne bacterial strains. These peptides have a narrow spectrum of activity and have shown potential as antibiotics. One specific peptide identified in the burdock root has similarities to a peptide with an antibiotic nature found in the *A. lappa L.* protein. Moreover, a dynamic dressing formulated with chitosan/alginate/genipin and produced through freeze-drying has been assessed for its anti-acne properties, indicating a potential mode of action for burdock in the treatment of acne [139].

### Alopecia

Alopecia is a common condition characterized by hair loss on the scalp. It can be caused by multiple factors, including autoimmune diseases, genetic factors, hormonal imbalances, and environmental influences. Diagnosis of alopecia is often based on clinical evaluation and may involve additional tests such as dermatoscopy and hormonal assays. Treatment options for alopecia include topical corticosteroids, immunomodulators, minoxidil, contact immunotherapy, and surgical interventions like hair transplantation [140]. *A. lappa L.* root contains active compounds like inulin, tannins, and essential oils that are believed to stimulate hair growth. These compounds help nourish the hair follicles and promote healthy hair growth. *A. lappa L.* root also has anti-inflammatory properties, which can help improve the condition of the scalp and reduce inflammation that may contribute to hair loss. Additionally, the antibacterial and antifungal effects of *A. lappa L.* root may help maintain a healthy scalp environment, which is essential for optimal hair growth. While scientific studies specifically focusing on the effects of *A. lappa L.* root on hair growth are limited, its traditional use and the presence of beneficial compounds suggest its potential to promote hair growth [85].

### Bone health

*A. lappa L.*, known colloquially as burdock, has been the subject of exploration regarding its potential advantages in promoting bone health. Arctigenin, an element extracted from the seeds of *A. lappa L.*, has demonstrated the ability to impede osteoclastogenesis, which is the mechanism responsible for bone resorption [141]. Arctigenin impedes the function of the nuclear factor of activated T-cells, cytoplasmic 1 (NFATc1), which serves as a pivotal transcription factor in osteoclastogenesis [138]. It inhibits both the calcineurin-dependent and osteoblastic cell-dependent NFATc1 pathways, crucial in regulating osteoclast differentiation and function [142]. Arctigenin is shown to possess inhibitory effects on the differentiation of osteoclast-like cells and their resorptive function [129]. Moreover, the root extract of *A. lappa L.* has demonstrated potential therapeutic benefits for metabolic-associated fatty liver disease and obesity, both of which are conditions known to have implications for bone health [143]. Nevertheless, additional investigation is necessary to comprehensively grasp the impact of *A. lappa L.* on bone health and its fundamental mechanisms.

### Anti-ulcer

**Fig. 13:**
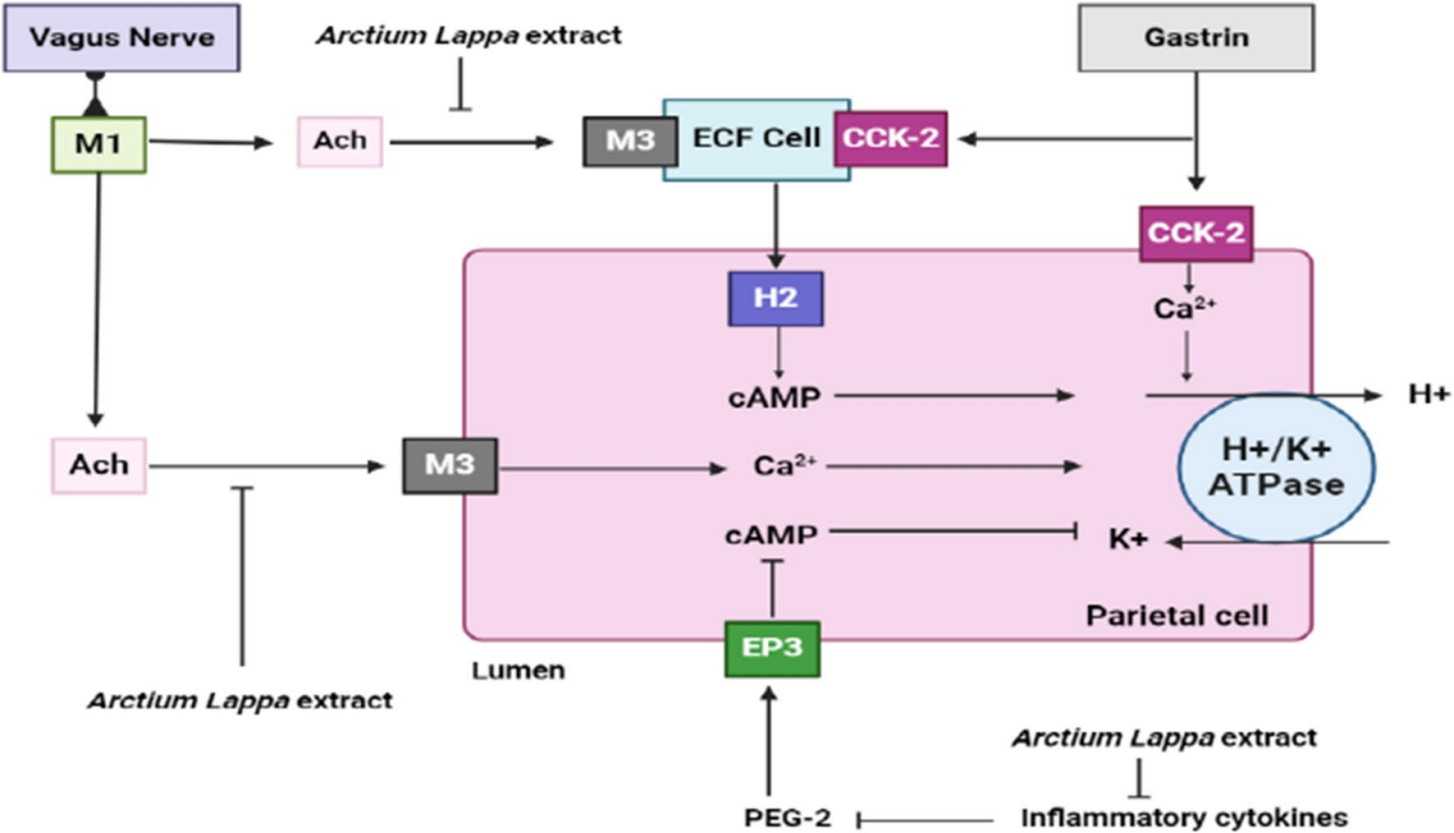
The schematic representation illustrates that the *A. lappa L.* extract hinders the attachment of acetylcholine to the M3 receptor on ECF cells, consequently inducing the liberation of histamine, which in turn binds to the H2 receptor, eliciting an elevation in cAMP within parietal cells that activates the H^+^/K^+^ ATPase. Moreover, the *A. lappa L.* extract also impedes the binding of acetylcholine to the M3 receptor present on parietal cells, leading to an augmentation in Ca^2+^ levels within the same cells, ultimately resulting in the stimulation of the H^+^/K^+^ ATPase. Furthermore, this extract demonstrates inhibition towards inflammatory cytokines that suppress PEG-2, thereby diminishing the levels of cAMP through interaction with the EP3 receptor. It is noteworthy that PEG-2 functions as a negative modulator of the H^+^/K^+^ ATPase. *Ach* acetylcholine, *cAMP* cyclic adenosine monophosphate, *EP*_*3*_ prostaglandin E2, *PEG-2* polyethylene glycol, *ECF* enterochromaffin-like cells, *CCK-2* cholecystokinin 2

Burdock has traditionally been utilized in China for both nourishment and medicinal purposes. It has been discovered to possess diverse therapeutic advantages, such as the facilitation of gastrointestinal mucosal mending in patients afflicted with ulcers. Several studies have shown that the burdock complex, including burdock as a component, can inhibit the adhesion of *Helicobacter pylori* to gastric epithelial cells and reduce levels of inflammatory markers. Burdock complex is a composite of burdock, angelica, gromwell, and sesame oil, commonly employed in traditional Chinese medicine. The research encompasses in vitro analyses on AGS cells (a human gastric adenocarcinoma cell line) and a clinical trial involving subjects with *Helicobacter pylori* infection. Burdock complex exhibits properties that are anti-inflammatory, antioxidant, and anti-adhesive in nature. Furthermore, the consumption of burdock complex by subjects infected with *Helicobacter pylori* resulted in enhanced antioxidant capacity, decreased urea breath test values, and diminished inflammatory markers, indicating a potential remedial effect on ulcers. The research encompasses in vitro analyses on AGS cells and a clinical trial involving subjects with *Helicobacter pylori* infection [144]. The alcoholic extraction derived from the root of the burdock plant exhibits a noteworthy anti-secretory effect through the partial suppression of H^+^ and K^+^-ATPase activity (Fig. 13).

The ethanolic extract derived from the root of *A. lappa L.* exhibited a reduction in both the volume and acidity of gastric secretion that had been elicited by bethanechol, histamine, and pentagastrin in vivo using stimulated pylorus-ligated rats. Furthermore, the extract from *A. lappa L.* root demonstrated a decrease in acetylcholine-induced contraction within gastric fundus strips. Interestingly, there was no observed effect on gastric relaxation induced by histamine following the administration of the *A. lappa L.* root extract [145]. The primary active component responsible for the anti-ulcer effect is arctigenin, which hinders the formation of gastric lesions caused by absolute ethanol and acetic acid in a manner that depends on the dosage, illustrating its anti-ulcerogenic properties. Arctigenin mitigates oxidative harm by reducing malondialdehyde levels and elevating superoxide dismutase levels in the bloodstream. Additionally, arctigenin demonstrates anti-inflammatory properties by reducing the levels of inflammatory mediators such as tumor necrosis factor-α, interleukin-6, interleukin-10, and C-reactive protein. This reduction in inflammatory damage further contributes to its anti-ulcer activity [145]. Similarly, chloroform extract of *A. lappa L.* root also shows gastroprotective activity [146].

### Aphrodisiac

The root extract of *A. lappa L.* has demonstrated aphrodisiac properties in male rats, enhancing various sexual behavior metrics including mount, intromission, and frequency of ejaculation [147]. The utilization of the extract has additionally led to a reduction in the time interval between ejaculation, while also increasing the frequencies of all components related to penile reflexes. The potential aphrodisiac effects of the root extract sourced from *A. lappa L.* could potentially be correlated with the existence of flavonoids, saponins, lignans, and alkaloids, which function through diverse central and peripheral mechanisms. The active constituents present in plant extracts, such as alkaloids, can enhance the dilation of blood vessels within the reproductive organs, consequently resulting in an improvement in sexual activity. Moreover, it can facilitate the process of penile erection by initiating vasodilation and relaxation of the penile corpus cavernosum through a mechanism that depends on nitric oxide. The aqueous extract obtained from the roots of *A. lappa L.* may boost the production and secretion of androgens, along with regulating the function of neurotransmitters, thereby further enhancing its aphrodisiac characteristics [7]. *A. lappa L.* extract demonstrates significant antioxidant properties, offering protection against ethanol-induced testicular injuries in rat models. The efficacy of *A. lappa L.* extract in mitigating testicular damage underscores its promising utilization in averting or ameliorating the adverse impacts of ethanol consumption on reproductive functions. Its antioxidant attributes enable the mitigation of reactive oxygen species production triggered by ethanol, surpassing the potency of ascorbic acid in this regard.

### Anti-viral

Porcine circovirus type 2(PCV2) is a viral agent that affects swine and is linked to Porcine Circovirus Associated Diseases. PCV2 can induce diverse clinical manifestations, including disorders related to respiratory and reproductive systems, along with immune suppression. The utilization of natural compounds derived from traditional Chinese medicines has proven effective in controlling PCV2. Marine and scutellarin are examples of such compounds, which have demonstrated antiviral properties by inhibiting the replication of the virus [148]. Arctigenin, a bioactive compound derived from the seeds of *A. lappa L.* exhibits notable immunomodulatory properties. It has been documented that Arctigenin can stimulate porcine alveolar macrophages by enhancing the production and release of cytokines, such as tumor necrosis factor-alpha and transforming growth factor beta-1, in a manner dependent on dosage. This stimulation is concomitant with a heightened phagocytic capacity of macrophages, suggesting their activation. The activation of macrophages by Arctigenin entails the Toll-like receptor 6 (TLR6)-NOX2 oxidase-MAPKs signaling pathway. Upon inhibition of the TLR6 receptor, the activation of NOX2 oxidase, cytokine secretion, and phagocytosis are diminished. Additionally, suppression of reactive oxygen species generation leads to a decrease in p38 and ERK1/2 phosphorylation, as well as cytokine secretion [149].

## Toxicity case of burdock roots

Only one recorded instance of consuming *A. lappa L.* tea has been documented. The case study reported in this paper outlines the manifestation of symptoms in a 26-year-old female patient who sought medical attention at the emergency department. The symptoms included visual impairment, urinary retention, xerostomia, and abnormal behavioral patterns, all of which occurred following the ingestion of an infusion made from *A. lappa L.* This occurrence is believed to be caused by the anticholinergic effects of atropine-like alkaloids. Anticholinergic toxicity can result in a range of symptoms, including dilated pupils, rapid heart rate, difficulty urinating, constipation, and cognitive decline. The production of *A. lappa L.* tea in the industrial setting involves various cultivation and processing techniques that lead to higher levels of alkaloids compared to traditional or homemade preparations. In mass spectrometry, it was found that commercially packaged *A. lappa L.* tea contains elevated amounts of an atropine-like alkaloid, which explains the anticholinergic symptoms experienced by the patient. The specific procedures involved in commercial packaging, including sourcing, processing, and packaging methods, may introduce or alter the alkaloid composition in the tea. Additional investigation is necessary to develop a thorough comprehension of the potential long-term health consequences and recommended consumption limits linked to *A. lappa L*. tea containing elevated levels of alkaloids [150].

## Future prospective

*A. lappa L.* exhibits promising potential for future applications. It has been noted that this substance exhibits considerable antibacterial properties, indicating its potential as a candidate for the creation of novel antibiotic medications [147]. Furthermore, the utilization of *A. lappa L.* has been observed in the production of composite films that possess antioxidant and antimicrobial characteristics. These films have the potential to serve as active packaging materials within the food industry sector [151]. Additionally, *A. lappa L.* extracts have showcased potential as complementary medicine against biofilm-forming uro-pathogens, effectively inhibiting biofilm formation and quorum-sensing-controlled cellular phenotypes. *A. lappa L.* has undergone evaluation for its antiviral impact against the hepatitis B virus and has demonstrated favorable outcomes, thereby justifying the need for further clinical trials [152]. The botanical extract has also manifested anti-inflammatory and anti-tumoral activities, signifying its potential utility in the treatment of acute inflammation and melanoma progression [153]. Furthermore, the complete genome of *A. lappa L.* has been sequenced, thereby conferring valuable insights into molecular identification, comparative genomics, and the identification of genes associated with *A. lappa L.* quality. In its entirety, these findings underscore the potential prospects of *A. lappa L.* across diverse domains, including medicine, agriculture, and genomics.

## Conclusion

*A. lappa L.* root harbors a multitude of active constituents dispersed throughout its various plant parts. These active constituents encompass a diverse array of polyphenols, lignans, flavonoids, volatile oils, and fructose-like inulin. The therapeutic effectiveness of these bioactive compounds spans a broad spectrum of health advantages, such as anticancer, antidiabetic, antiallergic, anti-inflammatory, antimicrobial, and numerous other beneficial properties. Furthermore, *A. lappa L.* exhibits neuroprotective properties possesses aphrodisiac qualities, and aids in weight loss. This botanical gem, owing to its robust antioxidant prowess, has been harnessed in various formulations such as *A. lappa L.* tea, powder, and capsules. Additionally, *A. lappa L.* is replete with vital vitamins, an assortment of minerals, and indispensable amino acids essential for bodily functions. *A. lappa L.* extract plays a crucial role in skin care products and functions as a preservative. It is imperative to emphasize the need for future research focusing on the standardization of *A. lappa L.* supplements. This is particularly important due to historical accounts of toxicity, and safety concerns for pregnant and lactating women, as well as children. Moreover, there is a necessity to investigate potential interactions between conventional medications and food, manage the withdrawal effects linked to burdock root, overcome the obstacles associated with traditional *A. lappa L.* formulations to enhance their therapeutic benefits, and design effective drug delivery systems for *A. lappa L.* nano-formulations.

## Data Availability

No data was used for the research described in the article.
